# Weighted 2D-kernel density estimations provide a new probabilistic measure for epigenetic age

**DOI:** 10.1186/s13059-025-03562-1

**Published:** 2025-04-22

**Authors:** Juan-Felipe Perez-Correa, Thomas Stiehl, Riccardo E. Marioni, Janie Corley, Simon R. Cox, Ivan G. Costa, Wolfgang Wagner

**Affiliations:** 1 Institute for Stem Cell Biology, RWTH Aachen University Medical School, Aachen, Germany; 2 Helmholtz Institute for Biomedical Engineering, RWTH Aachen University Medical School, Aachen, Germany; 3 Institute for Computational Biomedicine - Disease Modeling, RWTH Aachen University, Aachen, Germany; 4 Centre for Genomic and Experimental Medicine, Institute of Genetics and Cancer, University of Edinburgh, Edinburgh, UK; 5 Lothian Birth Cohorts, Department of Psychology, University of Edinburgh, Edinburgh, UK; 6 Institute for Computational Genomics, RWTH Aachen University Hospital, Aachen, Germany; 7 Center for Integrated Oncology, Aachen Bonn Cologne Düsseldorf (CIO ABCD), Aachen, Germany

**Keywords:** Epigenetic clocks, DNA methylation, Aging, Survival analysis, 2D density kernels

## Abstract

Epigenetic aging signatures provide insights into human aging, but traditional clocks rely on linear regression of DNA methylation levels, assuming linear trajectories. This study explores a non-parametric approach using 2D-kernel density estimation to determine epigenetic age. Our weighted model achieves similar predictive accuracy as conventional clocks and provides a variation score reflecting the inherent variability of age-related epigenetic changes within samples. This score is significantly increased in various diseases and associated with mortality risk in the Lothian Birth Cohort 1921. Thus, weighted 2D-kernel density estimation facilitates accurate epigenetic age predictions and offers an additional variable for biological age estimation.

**Supplementary Information:**

The online version contains supplementary material available at 10.1186/s13059-025-03562-1.

## Background

Aging is reflected by gains and losses of DNA methylation (DNAm) at specific sites in our genome and this can be used to determine donor age. Epigenetic clocks have become a central component of aging research [1]. Since they were first described [2, 3], epigenetic age-predictors have become more and more sophisticated and tailored toward specific applications [4]. First generation clocks are trained to correlate as highly as possible with chronological age, e.g., for applications in forensics [5–7]. Even in these clocks, the deviation of chronological and epigenetic age (delta age) is often indicative for all-cause mortality and affected by various diseases [8]. However, this phenomenon has been proven to decrease when the training sample size is large enough or when a correction for the white blood cells counts is applied [9]. To better reflect the biological age, multifactorial second-generation clocks have been described [10, 11]. In addition to age, they also integrate epigenetic indicators of other clinical parameters, such as blood counts, glucose levels, or blood pressure. Furthermore, third-generation clocks have been trained on large cohort studies and implement additional clinical measurements to better quantify the individual pace of aging [12].

Despite the wide range of epigenetic clocks that have already been established, most of them rely on the same approach: signatures of age-associated CG dinucleotides (CpGs) are preselected in a training dataset and then used for multivariable regressions under the assumption that there are linear changes of methylation with age. Particularly in childhood, age-associated DNAm changes follow a logarithmic pattern [13], which can be effectively addressed through non-linear corrections [5]—but the predictors still follow the assumption that there is a continuous trajectory—either linear or logarithmic. Besides, among older individuals it has been proven that the linear age predictors proposed by Horvath and Hannum regularly misestimate biological age, leading to predictions that are younger than the actual age [14]. This might partly be attributed to a survivor bias, wherein a reduced biological age in the elderly has facilitated the longevity of these individuals [15, 16]. However, this phenomenon may be also related to the nonlinear saturation effects observed in some aging-related CpG sites, where they converge to a stable value, i.e., 0 or 100% DNAm [17]. Recently, alternative non-linear epigenetic clocks have been described that are based on deep learning, e.g., by neuronal network frameworks in multidimensional space [18–20]. Furthermore, machine learning algorithms can be used to derive non-parametric epigenetic clocks based on Gaussian process regression models [21]. While these approaches theoretically use the full range of DNAm information of the training dataset, they are relatively complex, as they usually need a huge number of samples and computational power to work. For instance, Aliferi et al. developed a support vector machine-based clock by selecting markers from 51 different studies and more than 4000 patients [22], and the machine learning-driven clock created by de Lima Camillo and colleagues was trained and validated using 142 datasets and needs the full 20,318 CpGs that are shared among the Illumina Infinium HumanMethylation27, HumanMethylation450, and EPIC arrays as input [19]. Furthermore, all of these epigenetic clocks only provide one specific age-prediction for a given sample as a single output.

In this study, we introduce a novel methodology to build epigenetic clocks based on 2D kernel density estimation (KDE). For each individual CpG of the aging signature, the KDE creates a matrix that relates age and DNA methylation (DNAm) levels to corresponding density values, which are then translated into probabilities. Integration of these probabilities can then be used to determine the most likely age-prediction. Our KDE approach is non-parametric and does not require linear or logarithmic assumptions. We demonstrate that, particularly with a weighted KDE model, we can facilitate robust epigenetic age-predictions. Furthermore, the probability distribution provides insight into how consistent the age-associated DNAm is for a specific age-prediction in different sites of the genome. This variation score is a new complementary measure that may be useful to estimate biological age.

## Results

### Two-dimensional kernel distribution provides probabilistic epigenetic age estimates

**Fig. 1:**
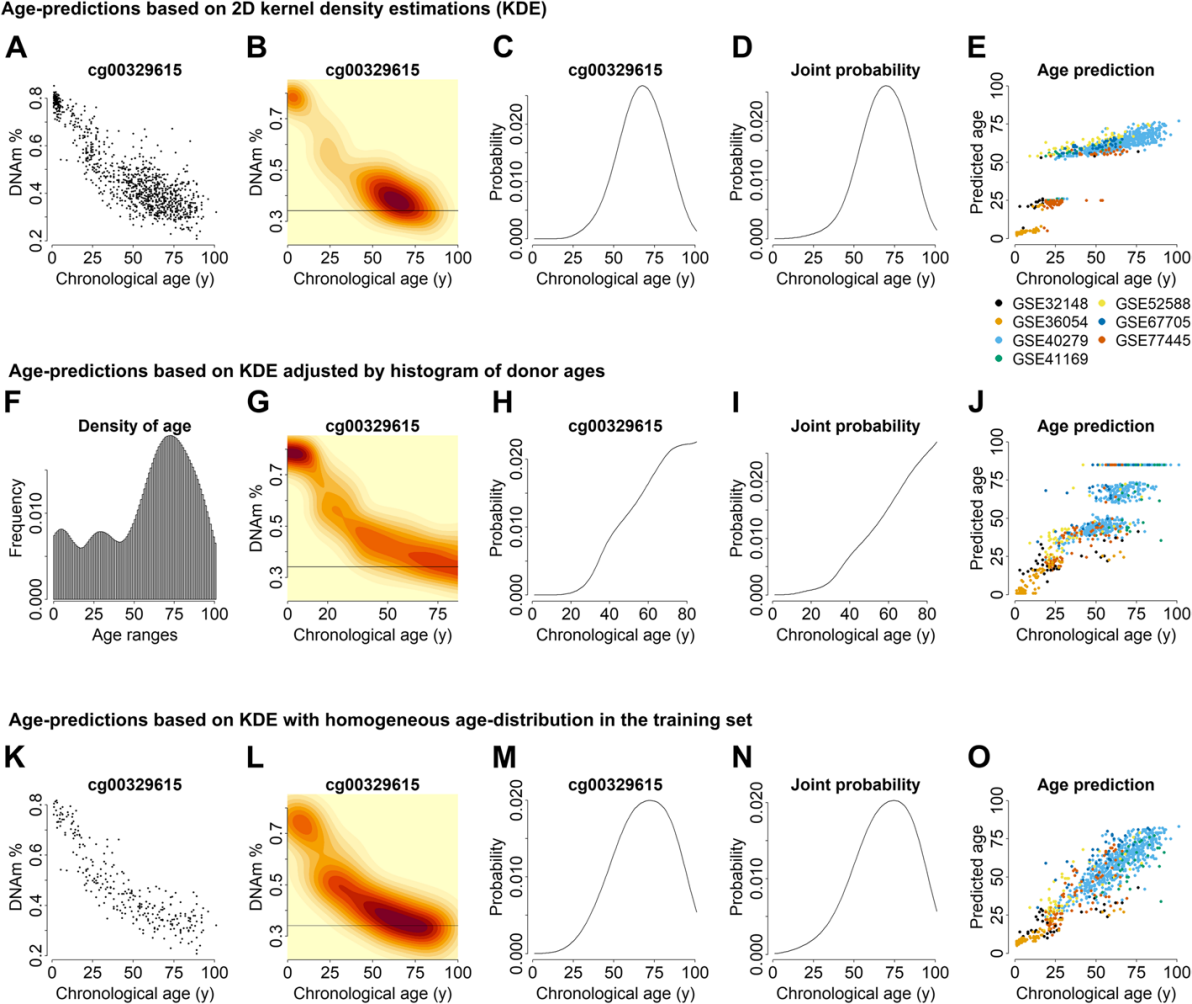
Construction of the probabilistic 2D kernel age predictor. **A** The association of DNA methylation (DNAm) with chronological age is exemplarily depicted for the CpG sites cg00329615 for all the samples in the training set. **B** 2D density kernel of the scatter plot depicted in **A**. The horizontal line exemplarily depicts the DNAm level of a given sample. **C** Density distribution as estimated for the probability of age-predictions for the sample depicted in B. **D** Joint probability for 27 age-associated CpGs for the same sample. **E** Performance of the methodology shown **A–D** for all the samples in the training set (color code indicates different studies of the training set). **F** Histogram of the ages in the training set. **G** Normalization of 2D kernels by age (divided by the corresponding value in the histogram). **H** Density distribution for the exemplary sample (line) in **G**. **I** Joint probability for 27 CpGs after normalization by age. **J** Performance of the methodology shown **F–I** for all the samples in the training set. **K** Alternatively, the training set was split in 5-year bins from 0 to 90 years (plus one bin of samples older than 90 years) and 15 samples from each bin were taken. The Scatter plot again depicts this selection for cg00329615. **L** 2D density kernel of **K**. **M** Density distribution for the sample in panel L. **N** Joint probability for 27 CpGs of the same sample. **O** Performance of the methodology shown **K–N** for all the samples in the training set

To develop epigenetic clocks based on KDE, we used DNAm datasets of 13 different studies on human peripheral blood that were separated into a training and a validation set. We arbitrarily preselected CpGs with the highest Pearson squared correlation with chronological age in the training set (R^2^ > 0.7: 27 CpGs, or R^2^ > 0.6: 491 CpGs). For each preselected CpG, the probability distribution of the age for a given DNAm level was determined by KDE and then combined into a joined probability estimation, where the age corresponding to the maximum probability was considered the predicted age of the sample. However, due to the heterogeneous distribution of donor ages, the predictions would be enriched at ages that are overrepresented in the training set (Fig. 1A–E).

Therefore, we normalized the KDE maps by the frequency of samples at each specific age. We excluded samples older than 85 years (*N* = 39), because their low frequency then resulted in high density in the 2D kernel maps. Adjustment of KDE by the histogram of donor ages provided more accurate predictions, but there were still clusters, indicating that the normalization now rather skewed for age-ranges with the highest density or with low number of samples (Fig. 1F–J).

To further reduce the effects of age distribution in the training set we subsequently divided the samples into 18 bins of 5-year intervals, ranging from 0 to 90 years, plus one 19^th^ bin with the samples older than 90 years. From each bin, 15 samples were selected to construct the 2D density kernels (Additional file 1: Figure S1). This resulted in 2D density kernels with a more even distribution of densities and epigenetic age predictions that correlated with chronological age (Fig. 1K–O). Thus, KDE can be used for a probabilistic approach for epigenetic age predictions.

### Improvement by weighted 2D kernel age-predictions

**Fig. 2:**
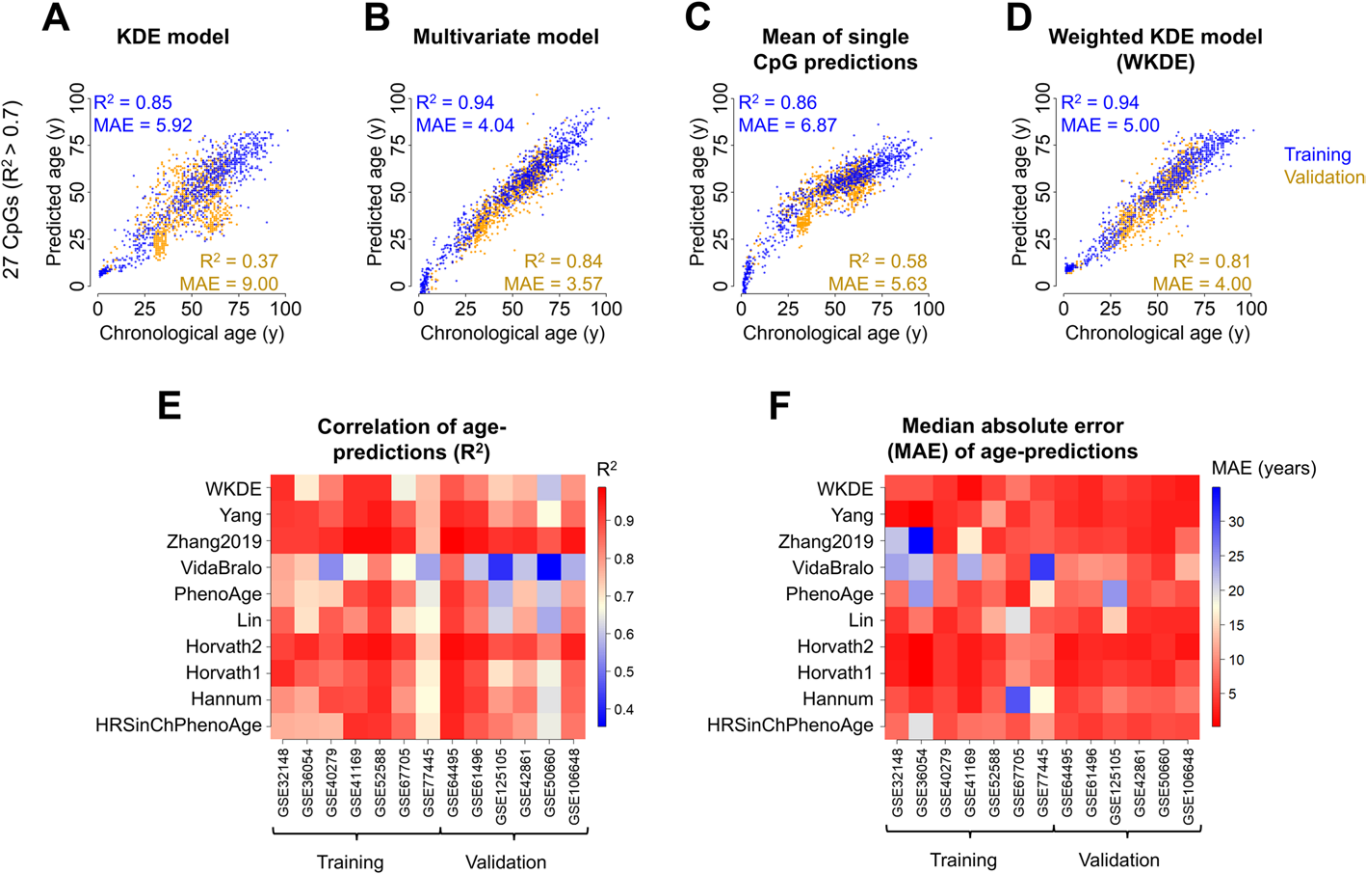
A weighted approach improves 2D kernel age predictions. **A** 2D kernel age prediction model was generated for 27 CpGs (R^2^ > 0.7 in the training set). The model was trained on a subset of samples from the training set with uniform age distribution. When we applied the model to the validation dataset, the predictions were not reliable. Pearson squared correlation R^2^ and median absolute error (MAE) are indicated. **B** The same CpGs were used to generate multivariate models based on the entire training set. **C** Alternatively, for each of the age-associated CpGs, a linear regression model was established to facilitate single CpG predictions. When we averaged these predictions, the performance was much lower than for the multivariable model. **D** The 2D kernel age-prediction model was further optimized by optimized weights for individual CpGs, which were determined by genetic algorithm optimization. **E** Heatmap of Pearson squared correlation (R^2^) between chronological and predicted age for WKDE and a set of widely used clocks, split in all datasets used for training and validation. **F** Heatmap of MAE for WKDE and the same set of clocks as in **E**, split in all datasets used for training and validation

When we initially tested our probabilistic kernel models based on either 27 or 491 CpGs, Pearson squared correlation with chronological age in the training set was R^2^ = 0.85 or 0.79, respectively, with a median absolute error (MAE) of 5 or 6 years. However, in the independent validation set, the correlations and precisions of age-predictions were much lower (27 CpG model: R^2^ = 0.37 and MAE = 9 years, Fig. 2A; 491 CpG model: R^2^ = 0.22 and MAE = 11 years, Additional file 1: Figure S2A), suggesting that there may be off-sets between different studies at individual CpGs, e.g., due to batch variation.

For comparison, we used the same set of age associated CpGs to generate multivariate regression models. This approach provided higher correlation with chronological age in the training set (27 CpG model: R^2^ = 0.94, Fig. 2B; 491 CpG model: R^2^ = 0.98, Additional file 1: Figure S2B). In fact, when creating multivariate models with the training set by varying the number of CpG sites, the highest correlation in the validation set was achieved with the top 125 CpGs (R^2^ = 0.92, Additional file 1: Figure S3A), while the highest precision in the validation set would have been achieved with the top 81 CpGs (MAE = 2.87 years, Additional file 1: Figure S3B), indicating that larger signatures are not always beneficial. In the independent validation set, the multivariable models with 27 or 491 CpGs revealed high correlation with age, too (27 CpG model R^2^ = 0.84, Fig. 2B; 491 CpG model R^2^ = 0.83, Additional file 1: Figure S2B). In contrast, when we just used the average of individual CpG predictions, the age-estimates were much less precise (27 CpG model: training: R^2^ = 0.86, validation: R^2^ = 0.58, Fig. 2C; 491 CpG model: training: R^2^ = 0.77, validation: R^2^ = 0.30, Additional file 1: Figure S2C). Furthermore, the ages of very young samples were overestimated due to exponential age-associated epigenetic changes in childhood [13]. These findings supported the notion that using conventional regression approaches as multivariable weighted approach was clearly advantageous.

Consequently, we have also adjusted our probabilistic kernel models to better weight the impact of individual CpGs. To this end, we used genetic algorithm optimization to identify the best weights for each CpG according to the cumulative error curves. This weighted kernel density estimate model (WKDE) improved predictions; also in the validation set (Fig. 2D, Additional file 1: Figure S2D)—particularly with the 27 CpG model, the predictions (R^2^ = 0.94 and MAE = 5 years in the training set; R^2^ = 0.81 and MAE = 4 years in the validation set) were now in a similar range as observed for the multivariable model. However, the 491 CpG model showed a very poor correlation in the validation set, which might be attributed to over-fitting of the weights and the increased amount features with lower age association. Therefore, we did not consider the 491 CpG WKDE model for further analysis.

### Benchmarking with other commonly used epigenetic clocks

Subsequently, we compared the performance of the 27 CpG WKDE model with other widely used epigenetic clocks in the field. To better test the applicability to independent datasets, we have done this analysis for each study of the training and validation sets. The WKDE predictor revealed better or similar correlations to many other clocks, while particularly the Zhang clock and Horvath Skin & Blood clock showed highest correlations (Fig. 2E). Furthermore, the WKDE model provided small median absolute errors for all datasets—apart from Yang clock and both Horvath clocks, other clocks revealed high MAEs of more than 15 years for at least one dataset (Fig. 2F). Thus, despite the small epigenetic signature of only 27 CpGs the WKDE model facilitated robust estimation of chronological age.

### Performance of the 27 CpG clock in purified cell types

To gain better insight into how the cellular composition affects age-predictions with our 27 CpG WKDE clock, we used a dataset with 6 purified cell types and provided chronological ages (GSE110554). Particularly for purified CD4 T cells, monocytes, neutrophils, and NK cells, the delta age was overall very low, whereas B cells were overestimated and CD8 T cells underestimated in age (Additional file 1: Figure S4A). These results were further validated with another dataset where the cell types were isolated form the same donors, but donor age was not provided (GSE224807). Again monocytes, granulocytes, and NK cells revealed similar age-predictions as for whole blood, whereas B cells were over-estimated, and T cells were predicted younger (Additional file 1: Figure S4B). These results demonstrate that while the 27 CpG WKDE clock can generally also be applied to purified cell types, the predictions may be influenced particularly by the composition of lymphocytes.

### Applicability of the WKDE method with targeted assays

To test if our new WKDE method was applicable to targeted epigenetic clocks, we used our previous published pyrosequencing data for nine age-associated CpGs in *FHL2*,* IGSF11*,* CCDC102B*,* MEIS1-AS3*,* ELOVL2*,* COL1 A1*,* PDE4 C*,* ASPA*, and* ITGA2B* [23]. After the 2-dimentional kernel calculation step and the genetic algorithm to assign the weight for each kernel, the 9 CpG model notably achieved a good correlation between chronological and predicted age and low prediction error in the validation set (R^2^ = 0.82, MAE = 5.33 weeks, Additional file 1: Figure S5), indicating that the approach is also suitable for targeted DNAm analysis, e.g. by pyrosequencing. 

### Heterogeneity of age-associated DNAm within a given sample

**Fig. 3:**
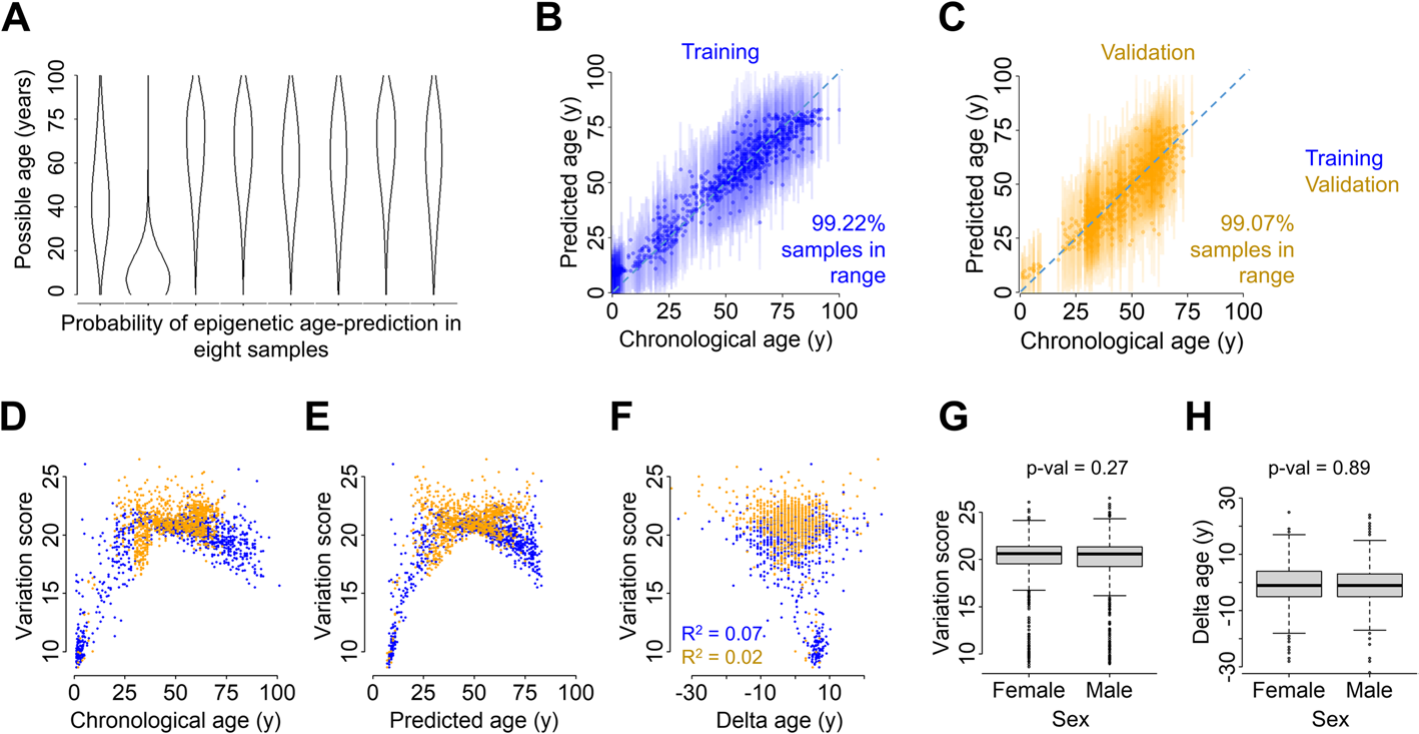
WKDE allows to measure heterogeneity in age-associated DNAm. **A** Possible age (*y*-axis) vs. probability (*x*-axis) for eight random samples from the training set. The thickness of the plot represents how probable is that a sample belongs to that age. **B** Chronological vs. predicted age in the training set measured with WKDE. Vertical lines depict predicted age ± variation score. **C** Same as in panel **B** for validation set. **D** Chronological age vs. variation score. **E** Predicted age vs. variation score. **F** Delta age vs. variation score. Variation score reflects almost no correlation with delta age measured with WKDE. **G** Boxplot of delta age vs. sex. Delta age distributes evenly across males and females (Wilcoxon rank sum test *P* = 0.27). **H** Boxplot of variation score vs. sex. Variation score distributes evenly across males and females (Wilcoxon rank sum test *P* = 0.89)

Our probabilistic approach allows to calculate the probability of the age of a given sample for every year from 0 to 100 (Fig. 3A). To estimate the disparity among the selected methylation sites, we calculate a variation score. This measure reflects the heterogeneity in age-associated DNAm within our 27 CpG signature, rather than the probability that the estimates of chronological age are correct. However, for most of the samples in training set (99.22% of total samples; Fig. 3B) and validation set (99.07% of total samples; Fig. 3C), the chronological age falls into this range (predicted age ± variation score). The variation score shows small correlations with chronological age (training: R^2^ = 0.36, validation: R^2^ = 0.33), epigenetic age (training: R^2^ = 0.37, validation: R^2^ = 0.24), and delta age (training: R^2^ = 0.07, validation: R^2^ = 0.02) and was highest at an age-range from 25 to 75 years (Figs. 3D–F). When we compared male and female samples, we did not observe any significant sex bias with regard to delta age (*P* = 0.27, Fig. 3G), or variation scores (*P* = 0.89; Fig. 3H).

### The variation score is significantly increased in several diseases

**Fig. 4:**
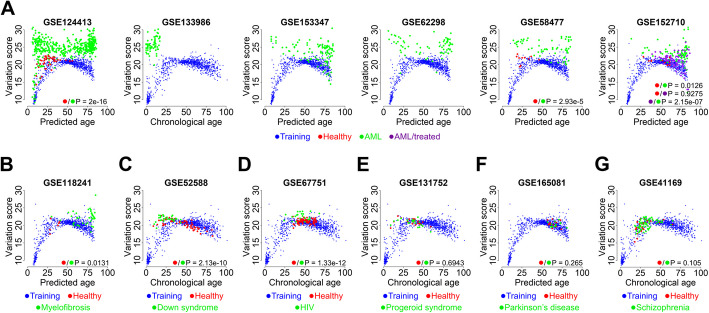
Variation score is significantly different in certain diseases. **A** The variation score was analyzed in six different datasets of acute myeloid leukemia (AML) and plotted in relation to donor age if provided or otherwise in relation to predicted age (with the 27 CpG WKDE model). Untreated AML samples (green), treated AML samples with azacitidine (purple), healthy controls from the same datasets if provided (red), and for comparison the training set of healthy controls are depicted. **B–G** The same analysis was performed for datasets of **B** myelofibrosis, **C** Down syndrome, **D** HIV, **E** progeroid syndromes, **F** Parkinson’s disease, and **G** schizophrenia. The indicated *P* values are related to the comparisons indicated by the corresponding color code

Epigenetic age-predictions can be impacted by diseases, particularly by hematopoietic malignancies [24]. Hence, we anticipated that the heterogeneity in age-associated DNAm changes, reflected by the variation score, may be affected in such diseases. To test this hypothesis, we used six publicly available DNAm datasets of healthy and diseased individuals for acute myeloid leukemia (AML). In fact, we observed pronounced differences in the distribution of variation score with age in all datasets, and when we compared diseased with available healthy individuals the difference was significant (GSE124413 *P* = 2e − 16, GSE58477, *P* = 2.93e − 5; GSE152710, *P* = 0.0126; Fig. 4A). Furthermore, the variation score declined upon therapy with azacytidine (GSE152710, *P* = 2.15e − 7) [25]. We have then exemplarily tested datasets of myelofibrosis, Down syndrome, HIV, progeroid syndromes, Parkinson’s disease, and schizophrenia (Additional file 1: Table S1), and calculated the corresponding variation score with our 27 CpG WKDE model. When we compared healthy and diseased individuals in these datasets, the variation score was significantly higher in myelofibrosis (*P* = 0.0131, Fig. 4B), Down syndrome (*P* = 2.13e − 10, Fig. 4C), and HIV (*P* = 1.33e − 12, Fig. 4D), whereas no significant difference was found in progeroid syndrome (*P* = 0.6943, Fig. 4E), Parkinson’s disease (*P* = 0.265, Fig. 4F), and schizophrenia (*P* = 0.105, Fig. 4G). Thus, high variation scores may be indicative for underlying diseases.

### Association of sample-intrinsic heterogeneity in epigenetic aging with all-cause mortality

**Fig. 5:**
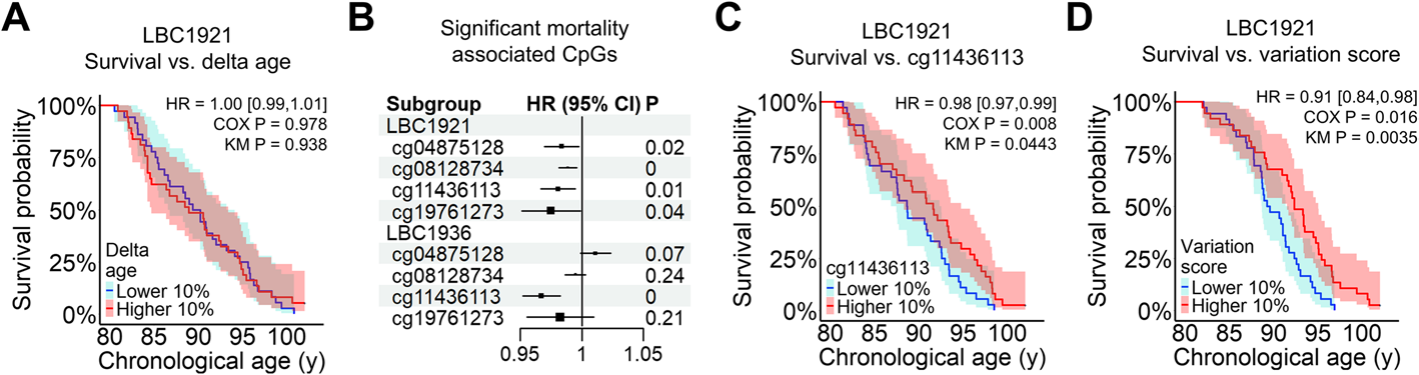
Variation score shows significant association with mortality. **A** The association of epigenetic age-predictions with all-cause mortality was analyzed in the Lothian Birth Cohort of 1921 (LBC1921). Kaplan-Meier survival curves are depicted for highest and lowest 10% delta ages. Cox regression model for all donors, adjusted for chronological age and sex, showed no significant effect of delta age in mortality risk (HR = 0.9998, 95% CI (0.988, 1.012), *P* = 0.978). **B** Individual Cox regressions for all the 27 CpGs in WKDE (adjusting for age and sex) revealed 4 significantly mortality-associated CpGs in the LBC1921. The cg11436113 was also significantly associated with mortality in the Lothian Birth Cohort of 1936 (LBC1936). **C** Kaplan-Meier survival curves for donors with highest and lowest 10% DNAm at cg11436113 in the LBC1921. Cox regression model, adjusted for chronological age and sex, shows that increase of 1% in the DNAm of cg11436113 is associated with a 1.94% decrease in mortality risk (95% CI (0.9665, 0.9949), *P* = 0.0080). **D** Kaplan-Meier survival curves for highest and lowest 10% variation score in the LBC1921. Cox regression model, adjusted for chronological age and sex, shows that increase of 1 unit in the variation score is associated with a 9.2% decrease in mortality risk (95% CI (0.8387, 0.9872), *P* = 0.0160)

It has been reported that all-cause mortality increases with accelerated epigenetic age in various clocks [8, 26]. To estimate if this also applies for our WKDE model, we analyzed methylation from the first waves of the LBC1921 and LBC1936 with subsequent mortality risk. Cox proportional hazards regression models for delta age vs. survival, adjusting for age and sex, revealed no significant association between delta age and mortality in the LBC1921 (*P* = 0.978, Fig. 5A) and in the LBC1936 (*P* = 0.415, Additional file 1: Figure S6A).

Subsequently, we tested if any of the individual CpG sites in the 27 CpG or 491 CpG models were associated with all-cause mortality, after adjustment for age and sex. Within the 491 CpGs, we found 25 mortality associated sites that overlap between LBC1921 and LBC1936 (Additional file 1: Figure S6B and Tables S2 and S3), with almost all of them being hypomethylated with aging. Out of the 27 CpG sites, four were significantly associated with mortality in the LBC1921 and one in the LBC1936 (Fig. 5B). Particularly cg11436113, which is located in an intergenic region, was highly associated with mortality in both cohorts (LBC1921: *P* < 0.008, Fig. 5C; LBC1936: *P* < 0.00003, Additional file 1: Figure S6C).

When testing for associations between variation score and mortality in the LBC1921, we observed that an increase of 1 unit in the variation score is associated with a 9.2% decrease in mortality risk (95% CI (0.8387, 0.9872), *P* = 0.0160, Figure 5D), after adjusting for sex and chronological age. The results remained significant when we adjusted for sex, chronological age, and delta age (*P* = 0.0159), indicating that the variation score might be an independent measure for biological aging. However, a significant association of the variation score with all-cause mortality was not observed in the LBC1936 (HR = 0.9894, 95% CI (0.8870, 1.1037), *P* = 0.849, Additional file 1: Figure S6D), which might partly be attributed to a lower number of deaths in this cohort.

Subsequently, we analyzed the association between variation score and mortality in a further adjusted model controlling for age, sex, delta age, and 22 additional parameters (Additional file 1: Table S4). Even this adjusted model indicated that a one-unit increase in the variation score is significantly associated with a 13.65% reduction in mortality risk within the LBC1921 (HR = 0.8635, 95% CI (0.7754,0.9617), *P* = 0.00754), which is still not reflected in the LBC1936 (HR = 1.0052, 95% CI (0.8765,1.1529), *P* = 0.9407).

## Discussion

In this study, we demonstrate that 2D kernel density estimation can be used for robust epigenetic age-predictions. Even with a very simplistic selection of 27 age-associated CpGs based on Pearson correlation with chronological age, we could demonstrate that WKDE models can provide similar precision as conventional epigenetic clocks. However, there are several ways how feature selections might be optimized in the future. Age-associated DNAm does not necessarily follow a strictly linear pattern [27]. This is also reflected by the 2D kernel density plots of the 27 CpGs, where almost all of them followed a logarithmic pattern. Thus, it might be advantageous to select the age-associated CpGs by Spearman correlation, albeit there is a very high overlap in age-associated CpGs selected with Pearson correlation [23]. Another approach might be to select CpGs based on the slope in single-CpG linear regressions to exclude CpGs that may reveal high correlation but only small absolute changes with age [28]. The selection of CpGs would be further improved by a larger collection of datasets, including newborns and centenarians. Our training set had a predominant emphasis on individuals of European ancestry and should better reflect ethnic diversity [29, 30]. To this end, we included datasets from USA (both European and Hispanic-Mexican ethnicity), the Netherlands, Denmark, Germany, the UK, and China in the training and/or validation sets. CpGs that show higher variation between cell types might be excluded from the predictors to reduce the impact of the cellular composition [23]. This might reduce the variation in age-predictions that we observed in B cells and T cells. Theoretically, automatic CpG selection approaches, such as ElasticNet [31], could be used to further optimize the selection of CpGs for WKDE—in analogy to the ElasticNet based conventional epigenetic clocks [5, 7]—but it would require an innovative automatic feature selection algorithm for matrices (taking also different possible weights into account) and this would require very high computational resources. Thus, in this study we introduce a new and very promising concept for constructing epigenetic clocks, rather than a fully optimized clock.

Nonetheless, WKDE method has some limitations. Predictions are only possible within the age-range that is covered by the training set and the estimated kernels—in our case, we can therefore not reliably predict ages above 100 years. The imbalance of different donor-ages in the training set was a major problem, which we could neither entirely corrected by normalization with the age-distribution, nor by a conditional density-based approach for the creation of the 2D density kernels [32]. We have therefore randomly reduced the training set for a uniform age-distribution—with the downside that not the entire training set was considered. In the future, alternative approaches based on resampling or machine learning might be further exploited to solve this limitation [33, 34]. Furthermore, our WKDE model was optimized with a genetic algorithm, which has shown several advantages over other non-linear optimization techniques, such as high efficiency, parallelization, and no need of derivative information [35]. However, other tuning parameters for the genetic algorithm or even non-linear optimization algorithms can be tested in subsequent studies.

Larger epigenetic signatures do not always increase the precision. When we only considered the top 27 CpGs in our training set to build the WKDE model, we observed a higher correlation with chronological age and lower median absolute error than using a less stringent pre-selection of 491 CpGs. This might be due to the more stringent selection of highly correlating CpGs and the greater number of CpGs increases the risk of overfitting of the genetic optimization algorithm to find optimal weighs [36]. In fact, the 491 WKDE model performed very poorly on the validation set, indicating that there was some overfitting.

Our model was specifically trained for human blood samples, while similar models might also be trained for other cell types or tissues in the future. There is a growing perception that even the heterogeneity of cells in blood can have major impact on epigenetic age-predictions with many conventional epigenetic aging clocks, and this may contribute to associations between clock-derived measures and age-related health outcomes [37]. For example, the association of epigenetic age acceleration and mortality found with Horvath and Hannum clocks seems to disappear when a correction for the white blood cell counts is performed [9]. Since we observed that purified B cells are predicted older whereas T cells are predicted younger, we anticipated that our signature will be impacted by cellular composition. Depending on the specific purpose of a study, it might be crucial to exclude CpGs with high variation between leukocyte subsets [23]. On the other hand, the 2D kernel density approach might also be applied for other epigenetic biomarkers, such as deconvolution of leukocytes in blood [38], or of cell types within a tissue [39, 40].

A big advantage of smaller signatures is that they can also be addressed by other methods for targeted DNAm analysis, such as pyrosequencing, digital PCR, or barcoded bisulfite amplicon sequencing [23]. In comparison to Illumina BeadChip technology, these approaches may facilitate faster and more cost-effective measurement. Furthermore, the measurements are independent of continuously changing microarray versions, which may anyway not be approved for clinical diagnostics [41]. In our previous work, we have measured 9 age-associated CpGs by pyrosequencing to derive an epigenetic aging clock with high accuracy based on pyrosequencing [23]. Notably, the same data can also be used to derive and validate a 9 CpG WKDE model for pyrosequencing data. This exemplifies that our approach is also applicable for targeted methods, particularly if the available training data is large enough.

Probabilistic approaches for epigenetic clocks have been described before. For example, we have previously described probabilistic epigenetic age predictions for individual sequencing reads, using the binary sequel of methylated and non-methylated CpGs in barcoded bisulfite amplicon sequencing data [23]. This method has been further developed for genome wide single-cell DNAm datasets [42]. In contrast, WKDE is not applicable to shallow sequencing data, but it provides density estimates for each CpG, a joint probability range for the sample and a variability score associated to it.

An important advantage of our approach is that the variation score provides a new measure for the intrinsic heterogeneity of age-associated DNAm changes. It is conceptionally somewhat related to a recently described “noise barometer” that is based on sums of standard deviations of DNAm at individual CpGs [43]. Notably, our variation score was clearly increased in malignancies, such as AML and myelofibrosis. Thus, even without knowing the chronological age, the combination of epigenetic age prediction and variation score can provide a “red flag” to initiate further diagnostic procedures. It was also striking that HIV, which has been shown to be associated with accelerated epigenetic clocks [44], might rather reflect increase heterogeneity of DNAm at age-associated CpGs.

Although association between accelerated age and mortality has been shown before [8, 26], we did not observe this with WKDE in the LBC1921, which might be due to the sample selection and the nature of the clock itself. We have intentionally only chosen samples from the initial waves of the LBC1921 and LBC1936 to isolate the effects of the variables under examination on mortality from the confounding factor of aging (which differs from the previously mentioned studies). It has to be noted, that our WKDE model was trained to correlate as closely as possible with chronological age and it is therefore conceivable that mortality-associated CpGs sites—such as cg11436113—were considered with a negative weight for age-predictions. Furthermore, WKDE employs a method of prediction that focuses on determining the age that maximizes the probability of a joint probability function for the age, rather than relying on a singular value. Hence, the biological age might still fall into the probability range of the age.

We have previously identified individual CpG sites from our 99-CpG clock and other epigenetic clocks that showed correlations with survival in both the LBC1921 and LBC1936 [45]—and most of these were hypomethylated with age [46]. Here, we showed an overlap of 25 methylation sites that are significantly associated with mortality in both cohorts, and again most of these CpGs become hypomethylated with age. Notably, these CpGs included cg06285727, cg06647068, cg08301612, and cg20813374, which were previously reported as all-cause mortality associated in a large-scale meta-EWAS study of six large cohorts [47]. Furthermore, 7 of them are located in genes *BST2*, *FKBP5*, *PRDX5*, *NWD1*, *FAM38 A*, and *NOD2*, which have previously shown high association between DNAm and aging [48–51], and we have already identified cg16363586 (located in gene *BST2*) as life expectancy associated site [45]. In addition, the CpG site that was identified as mortality-associated in both cohorts with the 27 CpG signature, cg11436113, has been reported as smoking and cancer related [52, 53].

While previous studies have discussed the link between age-related DNA methylation (DNAm) and mortality [54, 55], the connection between age-related DNAm heterogeneity and all-cause mortality was so far not addressed. Our results suggest that heterogeneity among age-associated methylation sites in the genome, measured as variability score, can provide additional insight into biological age. It may appear counterintuitive that here a lower variation score was associated with higher mortality, albeit very high variation scores can be indicative for other diseases, as mentioned above. This might be due to different pathophysiological mechanisms in aging and disease, or with the observation that particularly in the elderly the variation score declines again. Nevertheless, further validation is necessary to examine the potential application of the variability score to estimate life-expectancy and to explore possible association with specific diseases.

## Conclusions

Our study introduces weighted 2D-kernel density estimation (WKDE) to perform accurate epigenetic age predictions. Furthermore, we describe a variation score that may act as an additional parameter for evaluating biological age.

## Methods

### DNA methylation data

We compiled DNAm datasets of human peripheral blood samples of 13 different studies from Gene Expression Omnibus (GEO; Additional file 1: Table S5). We considered only samples that were classified as healthy or controls. Datasets were separated into a training set (7 Illumina HumanMethylation450 K array studies, 1029 samples, age range 1–101 years, 50.1% female) and an independent validation set (6 Illumina HumanMethylation450 K array studies, 980 samples, age range 2–79 years, 53.5% female). The data was processed in R 4.3.0. with the geoGEO function of the GEOquery package. To simplify the analysis and make it easily applicable to other groups, the preprocessed and normalized beta value matrices for each study were downloaded directly from the Gene Expression Omnibus (no additional background correction, normalization or quality control steps were performed, as they were already performed by the corresponding studies). CpGs in sex chromosomes and 6749 single-nucleotide polymorphism (SNP) probes (according to the HumanMethylation450 K v1.2 annotation) were filtered out.

### Epigenetic clock based on kernel density estimation

As a proof of concept that employing two-dimensional (2D) kernel estimations is feasible for accurate age predictions, we opted for one of the simplest feature selection approaches for age predictions: we have focused on the CpGs with highest linear correlation with chronological age in the training set, either 27 CpGs with R^2^ > 0.7 (Additional file 1: Table S6), or 491 CpGs with R^2^ > 0.6 (Additional file 1: Table S7). To generate KDE clocks based on these CpGs, we tested various alternative approaches. Initially, we used all 1029 samples of the training set to generate 2D kernel density plots of chronological age *versus* DNAm. Since this approach resulted in offsets of epigenetic age-predictions due to the heterogeneous age-distribution in the training set, we subsequently adjusted densities of the original kernel by the frequency of donor ages. To this end, we only considered samples ≤ 85 years to avoid a distortion for age-categories with very few samples. Alternatively, we tested conditional density resampled estimate of mutual information (DREMI) [32], which computes a two-dimensional kernel density estimate in cases where one variable, e.g., age, stochastically influences the other variable, e.g., DNAm. Even though this approach was meant to adjust the densities for the variations in DNA methylation linked to aging, it did not yield any enhancements in age prediction (data not shown). Finally, we randomly selected 15 samples of the training set for each 5-year bin from 0 to 90 years, and for one additional bin with samples older than 90 years. This resulted in a subset of 285 samples with uniform age distribution.

For each CpG, the kernels were generated with the function kde2d of the MASS R package, with 101 grid points, resulting in kernels with age on the *x*-axis (0–100 years), beta values on the *y*-axis (0 to 100% DNAm), and the density on the *z*-axis. Thus, each CpG kernel corresponds to a 3D matrix *K*, where ${K}_{i,j,k}$ represents the density value of the CpG $k$ for a sample with age $i$ and DNAm percentage $j$. An R object containing the 27 kernels, one for each of the 27 CpGs, can be found in Additional file 2 (.Rds). At this point, a preliminary probabilistic kernel age prediction for a sample can be done by:

$$predicted\ ag{e}_{preliminary}=\underset{i\in \left[\text{0,100}\right]}{\text{argmax}}\left(\sum_{k}{K}_{i,DNAm\left(k\right),k}\right)$$

where $k$ iterates through the selected CpGs and DNAm(*k*) is the DNAm percentage of the CpG *k* in the considered sample. For each CpG *k*, the row corresponding to the DNAm(*k*) is a vector of 101 densities, each of them corresponding to the years 0 to 100, respectively. After performing the summation of all these density vectors, the algorithm returns the age $i$, the one with the maximum cumulative density of the summation, as the final age.

To improve the performance of the predictor, a genetic algorithm was implemented to include a weight coefficient ${w}_{k}$ for each CpG $k$, with possible values in the range [− 10, 10]. For this, the function *minimize* from the R package *EmiR* was used, with a number of 150 initial conditions (parameter *population_size* = 150, meaning that 150 different vectors of 27 or 491 randomly assigned weights between − 10 and 10 were set as initial conditions at the start of the algorithm), 100 iterations (parameter *iterations* = 100, indicating that the algorithm will stop only after 100 iterations have been completed, and will return the best vector of 27 or 491 weights among the 100^th^ generation of descendants), a keep fraction of 0.4 (parameter *keep_fraction* = 0.4, so that 40% of the vectors survive for the next mating step), and a mutation rate of 0.1 (parameter *mutation_rate* = 0.1, implying a 10% probability of random modifications in the vectors of the offspring). The minimization function was set as:

$$\text{minimize}\left(\sum_{l}\left|ag{e}_{l}-\underset{i\in \left[\text{0,100}\right]}{\mathit{argmax}}\left(\sum_{k}{w}_{k}\cdot {K}_{i,DNA{m}_{l}\left(k\right),k}\right)\right|\right)$$

where $l$ iterates for all the samples in the training set, $ag{e}_{l}$ is the real age of the sample $l$ and $DNA{m}_{l}\left(k\right)$ is the DNAm percentage at site $k$ of the sample $l$. The genetic algorithm adjusts the weights of each CpG to minimize the sum of absolute differences between the predictions and the real ages, therefore finding the coefficients that minimize the total absolute error in the training set (Additional file 1: Tables S6 and S7). An R object containing the 27 weights for each CpG can be found in the Additional file 3 (.Rds). After finding the optimal weights, the final predictions can be calculated as:

$$predicted\ age=\underset{i\in \left[\text{0,100}\right]}{\mathit{argmax}}\left(\sum_{k}{w}_{k}\cdot {K}_{i,DNAm\left(k\right),k}\right)$$

In a similar way, a probability vector can be calculated. Every vector has one entry for each year (0 to 100), which quantifies how probable it is to observe the measured methylation value assuming that a person is of the respective age. Since each CpG is associated with a weight coefficient, we have to add the individual weighed vectors for all the CpGs, which ends up in a single merged vector. The vectors can be subsequently normalized to interpret the weighted sum as a probability, i.e., to ensure that the sum of probabilities is equal to 1. This results in the following formula for weighted 2D KDE (WKDE model):

$$probability\left(age\right)=\frac{\sum_{k=1}^{n}{w}_{k}\cdot {K}_{age,DNAm\left(k\right),k}}{\sum_{i=0}^{100}\left(\sum_{k=1}^{n}{w}_{k}\cdot {K}_{i,DNAm\left(k\right),k}\right)}$$

where $n$ is the number of the CpGs that the clock uses, ${w}_{k}$ is the weight of the CpG $k$, $DNAm(k)$ is the measured DNAm percentage of the CpG $k$ in the considered sample and ${K}_{age,DNA\left(k\right),k}$ is the value in the row $DNAm(k)$ and column $age$ of the density map of the CpG $k$.

### Epigenetic age predictions based on linear regression

To benchmark epigenetic age-predictions of our WKDE model with conventional approaches using the same subsets of CpGs (R^2^ > 0.6 or 0.7 in the training set, as indicated above), we established two alternative epigenetic clocks: (1) by multivariable linear regression using the *lm* function from stats package in R and the *predict* function to calculate age predictions for both training and validation sets (Additional file 1: Tables S6 and S7) and (2) by calculating the average of individual CpG age-predictions, whereby for each selected age-associated CpGs the slopes and intercepts between DNAm (beta values) and age were calculated with the *lm* and *apply* functions in R and the mean of the CpG-specific predictions was then used to estimate donor age (Additional file 1: Tables S6 and S7). Pearson squared correlation (R^2^) and median absolute error (MAE) between chronological and predicted age was calculated. Furthermore, we used the R package methylCIPHER [56] to compare the performance across the individual datasets used in training and validation sets with other commonly used epigenetic clocks: the Horvath clock [5], the Hannum clock [7], PhenoAge [10], retrained principal component PhenoAge [57], Horvath Skin & Blood epigenetic clock [58], Lin clock [45], Vidal-Bralo clock [59], Han clock [23], and Zhang clock [9].

### Age predictions in purified cell types

To estimate epigenetic age in purified leukocyte subsets, we used normalized matrices of beta values from two datasets: GSE110554 (30 samples after removing smokers and samples with missing information on chronological age) [60] and GSE224807 (415 samples after removing smokers, age-information is not provided) [61]. The data was downloaded from the GEO repository and processed in R 4.3.0. with the getGEO function of the GEOquery package, and no additional preprocessing or quality control steps were performed.

### Pyrosequencing-based WKDE clock

Pyrosequencing data of 80 healthy human whole blood samples (age range 19–73 years) was retrieved from a previous study [23]. The methylation values of 9 age-associated CpGs were used, and the samples were redistributed into training and validation sets so that the training set had a uniform age distribution (Additional file 1: Table S8). WKDE method was applied to build a clock with the 9 CpGs (both KDE calculation and genetic algorithm were performed as described before).

### Variation score

The variation score provides an estimate for how homogeneous the age-predictions for individual CpGs are. Thus, we focus on the probability vectors themselves, without considering the weights. For this, we used the R function *approxfun* with the non-weighted probability function to obtain probability values for all ages from 0 to 100 in steps of 0.1 years. The function *approxfun* performs a linear interpolation between given data points:

$$f\left(x\right)=approxfun\left(\frac{{\sum }_{k=1}^{n}{K}_{age,DNAm\left(k\right),k}}{{\sum }_{i=0}^{100}\left({\sum }_{k=1}^{n}{K}_{i,DNAm\left(k\right),k}\right)}\right)\left(x\right),where\ x\in \left\{\text{0,1},\dots ,\text{999,1000}\right\}$$

The call returns a vector with 1000 components, where the component x (which is between 0 and 1000) corresponds to the probability that the sample comes from an individual of age x/10. Subsequently, we were able to calculate mean, variance, and standard deviation of the probability function:

$$mean= 0.1\cdot \sum_{x=0}^{1000}f\left(x\right)\cdot x$$

$$variance=0.1\cdot \sum_{x=0}^{1000}f\left(x\right)\cdot {\left(0.1 x-mean\right)}^{2}$$

$$variation\ score=\sqrt{variance}$$

The R script with the functions to calculate predicted age, probability age function, and variation score with WKDE can be found in the additional file 4 (.R).

Finally, the Mann–Whitney-Wilcoxon test was used in R with the function *wilcox.test* to verify if there were differences in delta age (predicted − chronological age) by sex.

### Association between variation score and several diseases

The already preprocessed and normalized matrixes of beta values for the different studies (Additional file 1: Table S1) were directly downloaded from the GEO repositories. The data was processed in R 4.3.0. with the getGEO function of the GEOquery package, and no additional preprocessing or quality control steps were performed. When available, plots and statistics were performed with the chronological age of the samples; otherwise, the predicted age calculated with the 27 CpG WKDE model was used. The following repositories were used: GSE124413 [62], GSE133986 [63, 64], GSE153347 [65, 66], GSE62298 [67, 68], GSE58477 [69, 70], and GSE152710 [25, 71] for AML; GSE118241 [72, 73] for myelofibrosis, GSE52588 [74, 75] for Down syndrome, GSE67751 [76, 77] for HIV, GSE131752 [78, 79] for progeroid syndromes, GSE165081 [80, 81] for Parkinson’s disease, and GSE41169 [82, 83] for schizophrenia.

To assess whether there were significant differences in variation scores between healthy and diseased samples, ANOVA was utilized for each study individually. The variation score was designated as the response variable, with age (chronological or predicted), disease state, and the interaction of age and disease state included as factors. This methodology aimed to control for age, as the average age of the diseased and healthy samples differed in the majority of studies. The reported *p*-values for all the diseases correspond to the *p*-value of the factor “disease state” in the ANOVA.

### Mortality association in LBC1921 and LBC1936

The Lothian Birth Cohorts of 1921 (LBC1921) and 1936 (LBC1936) are follow-up studies of the Scottish Mental Surveys of participants born in 1921 and 1936, respectively. The study was initially set up to study determinants of non-pathological cognitive aging [84, 85] and peripheral blood samples were analyzed by 450 k Illumina BeadChips. Only samples from the first waves of the LBC1921 and LBC1936 were considered, to keep all the samples around the same chronological age (79.11 ± 0.59 years for LBC1921 and 69.56 ± 0.84 years for LBC1936)—thus, in these cohorts chronological age has little impact on the association with mortality analyses. All the raw data in the form of IDAT files were processed in R with the ENmix package. Quality control was performed, probes with detection *p*-values greater than 0.01 were filtered out, and probes and samples with more than 10% of missing values were excluded. ENmix_oob background correction [86], RELIC dye-bias correction [87], and RCP probe-type bias adjustment [88] were performed. To minimize the potential influence of fatal acute illnesses on the methylation measurements, deaths that occurred during the first 2 years of follow-up were excluded from the analysis. After the preprocessing filter for the first wave, a total of 374 samples from the LBC1921 and 721 from the LBC1936 remained.

Mortality status was ascertained from data linkage using dates of death (which were converted to age in days at death by the LBC research team) from the National Health Service Central Resister, provided by National Records of Scotland. Time of the events for the Cox models was defined as the interval between [*age in days at census wave1; age in days at death*]/365.25 in case of a death or [*age in days at census wave1; age in days at last census*]/365.25 in case of census. At time of last census, 367/374 (98.13%) and 285/721 (39.53%) had died. Delta age was calculated as predicted age (with the WKDE model) − chronological age. Cox proportional hazards regression models were performed (adjusted for age and sex), to assess the association between delta age and mortality and between variation score and mortality. Additional regression models were tested for delta age and variation score that also considered the following parameters as covariates: smoking status (never, former or current), alcohol consumption (units per week), counts in blood for basophils, eosinophils, neutrophils, leukocytes, monocytes, white cells and platelets, triglycerides, total serum cholesterol and vitamin B12 levels in blood, heart rate (information only available for LBC1921), sitting diastolic pressure, body mass index, grip strength, forced expiratory volume, telomere length, activity lifestyle score (LBC1921), physical activity level (LBC1936), self-perceived life quality, self-perceived health status, and self-perceived life enjoyment. Additional details regarding the cohorts and the different variables that were measured can be found in the Cohort Profile Update [85]. The statistical software R was used to conduct all analyses, employing the 'survival package for the Cox models. For fitting the Cox proportional hazards regression models, the function *coxph* was used. Significance of the relationship between Schoenfeld residuals and time for the basic adjusted (age and sex) and fully adjusted (all additional covariates mentioned above) models was calculated with the function *cox.zph*.

## Supplementary Information

Additional file 1. All supplemental figures and tables.
Additional files 2-5. Available at https://doi.org/10.5281/zenodo.11489551 [109].

## Data Availability

The following DNAm datasets, available in NCBI´s Gene Expression Omnibus repository, were analyzed in this study: DNAm from peripheral blood leukocytes from healthy children, GSE36054 [89, 90]; DNAm from peripheral blood samples from individuals with Crohn's disease and ulcerative colitis and healthy individuals, GSE32148 [91, 92]; DNAm from whole blood in schizophrenia and healthy patients, GSE41169 [ 82, 83 ]; DNAm from whole blood from healthy individuals, GSE77445 [93, 94]; DNAm from whole blood from healthy individuals and individuals with Down syndrome, GSE52588 [74, 75]; DNAm from whole blood from HIV infected and healthy individuals, GSE67705 [44, 95]; DNAm from whole blood from healthy individuals across a large age range, GSE40279 [7, 96]; DNAm from whole blood from individuals with syndrome X and healthy individuals, GSE64495 [97, 98]; DNAm from whole blood from birth-weight discordant twins, GSE61496 [99, 100]; DNAm from whole blood from depressed and control individuals, GSE125105 [101, 102]; DNAm from peripheral blood leukocytes from rheumatoid arthritis patients and healthy individuals, GSE42861 [ 103, 104 ]; DNAm from peripheral blood from current, former and never-smoker individuals, GSE50660 [105, 106]; DNAm from peripheral blood leukocytes from multiple sclerosis patients and healthy individuals, GSE106648 [107, 108]; DNAm from whole blood childhood acute myeloid leukemia, GSE124413 [62]; DNAm from bone from marrow in pediatric acute myeloid leukemia, GSE133986 [63, 64]; DNAm from bone marrow in patients with acute myeloid leukemia, GSE153347 [65, 66]; DNAm from whole blood in patients with acute myeloid leukemia, GSE62298 [67, 68]; DNAm from leukemic blast and bone marrow from patients with acute myeloid leukemia and healthy individuals, GSE58477 [69, 70]; DNAm from bone marrow from patients with high-risk myelodysplastic syndromes and secondary acute myeloid leukemia without treatment, patients treated with hypomethylating agents and healthy individuals, GSE152710 [25, 71]; DNAm from peripheral blood and bone marrow from patients with myelofibrosis and healthy individuals, GSE118241 [72, 73]; DNAm from whole blood from HIV positive and HIV negative individuals, GSE67751 [76, 77]; DNAm from whole blood in patients with progeroid syndromes and healthy individuals, GSE131752 [78, 79]; DNAm from whole blood from individual with Parkinson’s disease and healthy individuals, GSE165081 [80, 81]. Lothian Birth Cohort data are not publicly available due to containing information that could compromise participant consent and confidentiality, and are available only on request from the Lothian Birth Cohort Study, University of Edinburgh (https://www.ed.ac.uk/lothian-birth-cohorts/data-access-collaboration). Scripts and data for the WKDE method are provided in Additional file 2 (27 kernels calculated with our algorithm for the 27 CpG clock), Additional file 3 (27 weights for the 27 CpG clock), Additional file 4 (WKDE functions to calculate epigenetic age, probability age functions and variation score), and Additional file 5 (source code of the WKDE method as well as for reproducing the data analysis and main figures), are available on Zenodo repository under the MIT license at 10.5281/zenodo.11489551. [109].

## References

[1] Moqri M, Herzog C, Poganik JR, Ying K, Justice JN, Belsky DW, et al. Validation of biomarkers of aging. Nat Med. 2024;30(2):360–72. 38355974 10.1038/s41591-023-02784-9 PMC11090477

[2] Bocklandt S, Lin W, Sehl ME, Sanchez FJ, Sinsheimer JS, Horvath S, et al. Epigenetic predictor of age. PLoS ONE. 2011;6(6): e14821. 21731603 10.1371/journal.pone.0014821 PMC3120753

[3] Koch CM, Wagner W. Epigenetic-aging-signature to determine age in different tissues. Aging (Albany NY). 2011;3(10):1018–27. 22067257 10.18632/aging.100395 PMC3229965

[4] Horvath S, Raj K. DNA methylation-based biomarkers and the epigenetic clock theory of ageing. Nat Rev Genet. 2018;19(6):371–84. 29643443 10.1038/s41576-018-0004-3

[5] Horvath S. DNA methylation age of human tissues and cell types. Genome Biol. 2013;14(3156):1–19. 10.1186/gb-2013-14-10-r115 PMC4015143 24138928

[6] Weidner CI, Lin Q, Koch CM, Eisele L, Beier F, Ziegler P, et al. Aging of blood can be tracked by DNA methylation changes at just three CpG sites. Genome Biol. 2014;15(2): R24. 24490752 10.1186/gb-2014-15-2-r24 PMC4053864

[7] Hannum G, Guinney J, Zhao L, Zhang L, Hughes G, Sadda S, et al. Genome-wide methylation profiles reveal quantitative views of human aging rates. Mol Cell. 2013;49(2):459–367. 10.1016/j.molcel.2012.10.016 PMC3780611 23177740

[8] Marioni RE, Shah S, McRae AF, Chen BH, Colicino E, Harris SE, et al. DNA methylation age of blood predicts all-cause mortality in later life. Genome Biol. 2015;16(1):25. 25633388 10.1186/s13059-015-0584-6 PMC4350614

[9] Zhang Q, Vallerga CL, Walker RM, Lin T, Henders AK, Montgomery GW, et al. Improved precision of epigenetic clock estimates across tissues and its implication for biological ageing. Genome Med. 2019;11(1):54. 31443728 10.1186/s13073-019-0667-1 PMC6708158

[10] Levine ME, Lu AT, Quach A, Chen BH, Assimes TL, Bandinelli S, et al. An epigenetic biomarker of aging for lifespan and healthspan. Aging (Albany NY). 2018;10(4):573–91. 29676998 10.18632/aging.101414 PMC5940111

[11] Lu AT, Quach A, Wilson JG, Reiner AP, Aviv A, Raj K, et al. DNA methylation GrimAge strongly predicts lifespan and healthspan. Aging (Albany NY). 2019;11(2):303–27. 30669119 10.18632/aging.101684 PMC6366976

[12] Belsky DW, Caspi A, Corcoran DL, Sugden K, Poulton R, Arseneault L, et al. DunedinPACE, a DNA methylation biomarker of the pace of aging. Elife. 2022;11: e73420. 35029144 10.7554/eLife.73420 PMC8853656

[13] Alisch RS, Barwick BG, Chopra P, Myrick LK, Satten GA, Conneely KN, et al. Age-associated DNA methylation in pediatric populations. Genome Res. 2012;22(4):623–32. 22300631 10.1101/gr.125187.111 PMC3317145

[14] El Khoury LY, Gorrie-Stone T, Smart M, Hughes A, Bao Y, Andrayas A, et al. Systematic underestimation of the epigenetic clock and age acceleration in older subjects. Genome Biol. 2019;20(1):283. 31847916 10.1186/s13059-019-1810-4 PMC6915902

[15] Simpson DJ, Chandra T. Epigenetic age prediction. Aging Cell. 2021;20(9): e13452. 34415665 10.1111/acel.13452 PMC8441394

[16] Bell CG, Lowe R, Adams PD, Baccarelli AA, Beck S, Bell JT, et al. DNA methylation aging clocks: challenges and recommendations. Genome Biol. 2019;20(1):249. 31767039 10.1186/s13059-019-1824-y PMC6876109

[17] Crofts SJC, Latorre-Crespo E, Chandra T. DNA methylation rates scale with maximum lifespan across mammals. Nature Aging. 2024;4(1):27–32. 38049585 10.1038/s43587-023-00535-6 PMC10798888

[18] Li A, Mueller A, English B, Arena A, Vera D, Kane AE, et al. Novel feature selection methods for construction of accurate epigenetic clocks. PLoS Comput Biol. 2022;18(8): e1009938. 35984867 10.1371/journal.pcbi.1009938 PMC9432708

[19] de Lima Camillo LP, Lapierre LR, Singh R. A pan-tissue DNA-methylation epigenetic clock based on deep learning. npj Aging. 2022;8(1):4.

[20] Galkin F, Mamoshina P, Kochetov K, Sidorenko D, Zhavoronkov A. DeepMAge: A Methylation aging clock developed with deep learning. Aging Dis. 2021;12(5):1252–62. 34341706 10.14336/AD.2020.1202 PMC8279523

[21] Varshavsky M, Harari G, Glaser B, Dor Y, Shemer R, Kaplan T. Accurate age prediction from blood using a small set of DNA methylation sites and a cohort-based machine learning algorithm. Cell Rep Methods. 2023;3(9): 100567. 37751697 10.1016/j.crmeth.2023.100567 PMC10545910

[22] Aliferi A, Sundaram S, Ballard D, Freire-Aradas A, Phillips C, Lareu MV, et al. Combining current knowledge on DNA methylation-based age estimation towards the development of a superior forensic DNA intelligence tool. Forensic Sci Int Genet. 2022;57: 102637. 34852982 10.1016/j.fsigen.2021.102637

[23] Han Y, Franzen J, Stiehl T, Gobs M, Kuo CC, Nikolic M, et al. New targeted approaches for epigenetic age predictions. BMC Biol. 2020;18(1):71. 32580727 10.1186/s12915-020-00807-2 PMC7315536

[24] Lin Q, Wagner W. Epigenetic Aging Signatures Are Coherently Modified in Cancer. PLoS Genet. 2015;11(6): e1005334. 26110659 10.1371/journal.pgen.1005334 PMC4482318

[25] Cabezón M, Malinverni R, Bargay J, Xicoy B, Marcé S, Garrido A, et al. Different methylation signatures at diagnosis in patients with high-risk myelodysplastic syndromes and secondary acute myeloid leukemia predict azacitidine response and longer survival. Clin Epigenetics. 2021;13(1):9. 33446256 10.1186/s13148-021-01002-y PMC7809812

[26] Marioni RE, Shah S, McRae AF, Ritchie SJ, Muniz-Terrera G, Harris SE, et al. The epigenetic clock is correlated with physical and cognitive fitness in the Lothian Birth Cohort 1936. Int J Epidemiol. 2015;44(4):1388–96. 25617346 10.1093/ije/dyu277 PMC4588858

[27] Vershinina O, Bacalini MG, Zaikin A, Franceschi C, Ivanchenko M. Disentangling age-dependent DNA methylation: deterministic, stochastic, and nonlinear. Sci Rep. 2021;11(1):9201. 33911141 10.1038/s41598-021-88504-0 PMC8080842

[28] Perez-Correa JF, Tharmapalan V, Geiger H, Wagner W. Epigenetic clocks for mice based on age-associated regions that are conserved between mouse strains and human. Front Cell Dev Biol. 2022;10: 902857. 35721486 10.3389/fcell.2022.902857 PMC9204067

[29] Breeze CE, Beck S, Berndt SI, Franceschini N. The missing diversity in human epigenomic studies. Nat Genet. 2022;54(6):737–9. 35681055 10.1038/s41588-022-01081-4 PMC9832920

[30] Becker J, Böhme P, Reckert A, Eickhoff SB, Koop BE, Blum J, et al. Evidence for differences in DNA methylation between Germans and Japanese. Int J Legal Med. 2022;136(2):405–13. 34739581 10.1007/s00414-021-02736-3 PMC8847189

[31] Zou H, Hastie T. Regularization and Variable Selection Via the Elastic Net. J R Stat Soc Ser B Stat Methodol. 2005;67(2):301–20.

[32] Krishnaswamy S, Spitzer MH, Mingueneau M, Bendall SC, Litvin O, Stone E, et al. Systems biology. Conditional density-based analysis of T cell signaling in single-cell data. Science. 2014;346(6213):1250689. 25342659 10.1126/science.1250689 PMC4334155

[33] Wu O. Rethinking class imbalance in machine learning. 2023:[arXiv:2305.03900 p.]. Available from: https://ui.adsabs.harvard.edu/abs/2023arXiv230503900W.

[34] Yang J, El-Bouri R, O’Donoghue O, Lachapelle AS, Soltan AAS, Eyre DW, et al. Deep reinforcement learning for multi-class imbalanced training: applications in healthcare. Mach Learn. 2023;113:2655–74. 38708086 10.1007/s10994-023-06481-z PMC11065699

[35] Katoch S, Chauhan SS, Kumar V. A review on genetic algorithm: past, present, and future. Multimedia Tools and Applications. 2021;80(5):8091–126. 33162782 10.1007/s11042-020-10139-6 PMC7599983

[36] Hawkins DM. The Problem of Overfitting. J Chem Inf Comput Sci. 2004;44(1):1–12. 14741005 10.1021/ci0342472

[37] Jonkman TH, Dekkers KF, Slieker RC, Grant CD, Ikram MA, van Greevenbroek MMJ, et al. Functional genomics analysis identifies T and NK cell activation as a driver of epigenetic clock progression. Genome Biol. 2022;23(1):24. 35031073 10.1186/s13059-021-02585-8 PMC8759260

[38] Hubens WHG, Maie T, Schnitker M, Bocova L, Puri D, Wessiepe M, et al. Targeted DNA methylation analysis facilitates leukocyte counts in dried blood samples. Clin Chem. 2023;69(11):1283–94. 37708296 10.1093/clinchem/hvad143

[39] Moss J, Magenheim J, Neiman D, Zemmour H, Loyfer N, Korach A, et al. Comprehensive human cell-type methylation atlas reveals origins of circulating cell-free DNA in health and disease. Nat Commun. 2018;9(1):5068. 30498206 10.1038/s41467-018-07466-6 PMC6265251

[40] Schmidt M, Maié T, Dahl E, Costa IG, Wagner W. Deconvolution of cellular subsets in human tissue based on targeted DNA methylation analysis at individual CpG sites. BMC Biol. 2020;18:178. 33234153 10.1186/s12915-020-00910-4 PMC7687708

[41] Wagner W. How to translate DNA methylation biomarkers into clinical practice. Frontiers in Cell and Developmental Biology. 2022;10: 854797. 35281115 10.3389/fcell.2022.854797 PMC8905294

[42] Trapp A, Kerepesi C, Gladyshev VN. Profiling epigenetic age in single cells. Nat Aging. 2021;1(1):1189–201. 36211119 10.1038/s43587-021-00134-3 PMC9536112

[43] Mei X, Blanchard J, Luellen C, Conboy MJ, Conboy IM. Fail-tests of DNA methylation clocks, and development of a noise barometer for measuring epigenetic pressure of aging and disease. Aging (Albany NY). 2023;15(17):8552–75. 37702598 10.18632/aging.205046 PMC10522373

[44] Gross Andrew M, Jaeger Philipp A, Kreisberg Jason F, Licon K, Jepsen Kristen L, Khosroheidari M, et al. Methylome-wide analysis of chronic HIV infection reveals five-year increase in biological age and epigenetic targeting of HLA. Mol Cell. 2016;62(2):157–68. 27105112 10.1016/j.molcel.2016.03.019 PMC4995115

[45] Lin Q, Weidner CI, Costa IG, Marioni RE, Ferreira MR, Deary IJ, et al. DNA methylation levels at individual age-associated CpG sites can be indicative for life expectancy. Aging (Albany NY). 2016;8(2):394–401. 26928272 10.18632/aging.100908 PMC4789590

[46] Zhang Y, Hapala J, Brenner H, Wagner W. Individual CpG sites that are associated with age and life expectancy become hypomethylated upon aging. Clin Epigenetics. 2017;9:9. 28168006 10.1186/s13148-017-0315-9 PMC5288846

[47] Bernabeu E, McCartney DL, Gadd DA, Hillary RF, Lu AT, Murphy L, et al. Refining epigenetic prediction of chronological and biological age. Genome Medicine. 2023;15(1):12. 36855161 10.1186/s13073-023-01161-y PMC9976489

[48] Beach SRH, Ong ML, Lei MK, Carter SE, Simons RL, Gibbons FX, et al. Methylation of FKBP5 is associated with accelerated DNA methylation ageing and cardiometabolic risk: replication in young-adult and middle-aged Black Americans. Epigenetics. 2022;17(9):982–1002. 34533092 10.1080/15592294.2021.1980688 PMC9487733

[49] Acevedo N, Reinius LE, Vitezic M, Fortino V, Soderhall C, Honkanen H, et al. Age-associated DNA methylation changes in immune genes, histone modifiers and chromatin remodeling factors within 5 years after birth in human blood leukocytes. Clin Epigenetics. 2015;7(1):34. 25874017 10.1186/s13148-015-0064-6 PMC4396570

[50] Florath I, Butterbach K, Muller H, Bewerunge-Hudler M, Brenner H. Cross-sectional and longitudinal changes in DNA methylation with age: an epigenome-wide analysis revealing over 60 novel age-associated CpG sites. Hum Mol Genet. 2014;23(5):1186–201. 24163245 10.1093/hmg/ddt531 PMC3919014

[51] Marttila S, Kananen L, Hayrynen S, Jylhava J, Nevalainen T, Hervonen A, et al. Ageing-associated changes in the human DNA methylome: genomic locations and effects on gene expression. BMC Genomics. 2015;16(1):179. 25888029 10.1186/s12864-015-1381-z PMC4404609

[52] Wang C, Amini H, Xu Z, Peralta AA, Yazdi MD, Qiu X, et al. Long-term exposure to ambient fine particulate components and leukocyte epigenome-wide DNA Methylation in older men: the Normative Aging Study. Environ Health. 2023;22(1):54. 37550674 10.1186/s12940-023-01007-5 PMC10405403

[53] Vermeulen R, Bodinier B, Dagnino S, Wada R, Wang X, Silverman D, et al. A prospective study of smoking-related white blood cell DNA methylation markers and risk of bladder cancer. Eur J Epidemiol. 2024;39(4):393–407. 38554236 10.1007/s10654-024-01110-y PMC11101379

[54] Lund JB, Li S, Baumbach J, Svane AM, Hjelmborg J, Christiansen L, et al. DNA methylome profiling of all-cause mortality in comparison with age-associated methylation patterns. Clin Epigenetics. 2019;11(1):23. 30736859 10.1186/s13148-019-0622-4 PMC6368749

[55] Colicino E, Marioni R, Ward-Caviness C, Gondalia R, Guan W, Chen B, et al. Blood DNA methylation sites predict death risk in a longitudinal study of 12, 300 individuals. Aging (Albany NY). 2020;12(14):14092–124. 32697766 10.18632/aging.103408 PMC7425458

[56] Thrush KL, Higgins-Chen AT, Liu Z, Levine ME. R methylCIPHER: a methylation clock investigational package for hypothesis-driven evaluation & research. bioRxiv. 2022.07.13.499978 [Preprint]. 2022 [cited on October 10 2024]. Available from https://www.biorxiv.org/content/10.1101/2022.07.13.499978v1.

[57] Higgins-Chen AT, Thrush KL, Wang Y, Minteer CJ, Kuo P-L, Wang M, et al. A computational solution for bolstering reliability of epigenetic clocks: implications for clinical trials and longitudinal tracking. Nature Aging. 2022;2(7):644–61. 36277076 10.1038/s43587-022-00248-2 PMC9586209

[58] Horvath S, Oshima J, Martin GM, Lu AT, Quach A, Cohen H, et al. Epigenetic clock for skin and blood cells applied to Hutchinson Gilford Progeria Syndrome and ex vivo studies. Aging (Albany NY). 2018;10(7):1758–75. 30048243 10.18632/aging.101508 PMC6075434

[59] Vidal-Bralo L, Lopez-Golan Y, Gonzalez A. Simplified assay for epigenetic age estimation in whole blood of adults. Front Genet. 2016;7:126. 27471517 10.3389/fgene.2016.00126 PMC4943959

[60] Salas LA, Koestler DC, Butler RA, Hansen HM, Wiencke JK, Kelsey KT, et al. An optimized library for reference-based deconvolution of whole-blood biospecimens assayed using the Illumina HumanMethylationEPIC BeadArray. Genome Biol. 2018;19(1):64. 29843789 10.1186/s13059-018-1448-7 PMC5975716

[61] Wang X, Campbell MR, Cho H-Y, Pittman GS, Martos SN, Bell DA. Epigenomic profiling of isolated blood cell types reveals highly specific B cell smoking signatures and links to disease risk. Clin Epigenetics. 2023;15(1):90. 37231515 10.1186/s13148-023-01507-8 PMC10211291

[62] Farrar J, Triche T. GSE124413. Gene Expression Omnibus. 2020. https://www.ncbi.nlm.nih.gov/geo/query/acc.cgi?acc=GSE124413.

[63] Yamato G, Kawai T, Shiba N, Ikeda J, Hara Y, Ohki K, et al. Genome-wide DNA methylation analysis in pediatric acute myeloid leukemia. Blood Adv. 2022;6(11):3207–19. 35008106 10.1182/bloodadvances.2021005381 PMC9198913

[64] Yamato G, Kawai T, Shiba N, Hara Y, Ohki K, Kaburagi T, et al. GSE133986. Gene Expression Omnibus. 2022. https://www.ncbi.nlm.nih.gov/geo/query/acc.cgi?acc=GSE133986.

[65] Wang F, Morita K, DiNardo CD, Furudate K, Tanaka T, Yan Y, et al. Leukemia stemness and co-occurring mutations drive resistance to IDH inhibitors in acute myeloid leukemia. Nat Commun. 2021;12(1):2607. 33972549 10.1038/s41467-021-22874-x PMC8110775

[66] Wang F, Takahashi K. GSE153347. Gene Expression Omnibus. 2021. https://www.ncbi.nlm.nih.gov/geo/query/acc.cgi?acc=GSE153347.

[67] Ferreira HJ, Heyn H, Vizoso M, Moutinho C, Vidal E, Gomez A, et al. DNMT3A mutations mediate the epigenetic reactivation of the leukemogenic factor MEIS1 in acute myeloid leukemia. Oncogene. 2016;35(23):3079–82. 26434589 10.1038/onc.2015.359 PMC4705435

[68] Esteller M, Heyn H. GSE62298. Gene Expression Omnibus. 2015. https://www.ncbi.nlm.nih.gov/geo/query/acc.cgi?acc=GSE62298.

[69] Qu Y, Lennartsson A, Gaidzik VI, Deneberg S, Karimi M, Bengtzén S, et al. Differential methylation in CN-AML preferentially targets non-CGI regions and is dictated by DNMT3A mutational status and associated with predominant hypomethylation of HOX genes. Epigenetics. 2014;9(8):1108–19. 24866170 10.4161/epi.29315 PMC4164496

[70] Qu Y, Lennartsson A, I.Gaidzik V, Deneberg S, Karimi M, Bengtzén S, et al. GSE58477. Gene Expression Omnibus. 2014. https://www.ncbi.nlm.nih.gov/geo/query/acc.cgi?acc=GSE58477.

[71] Cabezón M, Malinverni R, Bargay J, Xicoy B, Marcé S, Garrido A, et al. GSE152710. Gene Expression Omnibus. 2021. https://www.ncbi.nlm.nih.gov/geo/query/acc.cgi?acc=GSE152710.

[72] Martínez-Calle N, Pascual M, Ordoñez R, Enériz ESJ, Kulis M, Miranda E, et al. Epigenomic profiling of myelofibrosis reveals widespread DNA methylation changes in enhancer elements and ZFP36L1 as a potential tumor suppressor gene that is epigenetically regulated. Haematologica. 2019;104(8):1572–9. 30655376 10.3324/haematol.2018.204917 PMC6669145

[73] Martínez-Calle N, Pascual M, Ordoñez R, Agirre X, Prosper F. GSE118241. Gene expression omnibus. 2019. https://www.ncbi.nlm.nih.gov/geo/query/acc.cgi?acc=GSE118241.

[74] Bacalini MG, Boattini A, Gentilini D, Giampieri E, Pirazzini C, Giuliani C, et al. A meta-analysis on age-associated changes in blood DNA methylation: results from an original analysis pipeline for Infinium 450k data. Aging (Albany NY). 2015;7(2):97–109. 25701668 10.18632/aging.100718 PMC4359692

[75] Bacalini M, Gentilini D, Boattini A, Giampieri E, Pirazzini C, Giuliani C, et al. GSE52588. Gene Expression Omnibus. 2015. https://www.ncbi.nlm.nih.gov/geo/query/acc.cgi?acc=GSE52588.

[76] Horvath S, Levine AJ. HIV-1 infection accelerates age according to the epigenetic clock. J Infect Dis. 2015;212(10):1563–73. 25969563 10.1093/infdis/jiv277 PMC4621253

[77] Horvath S, Levine A. GSE67751. Gene Expression Omnibus. 2015. https://www.ncbi.nlm.nih.gov/geo/query/acc.cgi?acc=GSE67751.

[78] Maierhofer A, Flunkert J, Oshima J, Martin GM, Poot M, Nanda I, et al. Epigenetic signatures of Werner syndrome occur early in life and are distinct from normal epigenetic aging processes. Aging Cell. 2019;18(5): e12995. 31259468 10.1111/acel.12995 PMC6718529

[79] Maierhofer A, Flunkert J, Oshima J, Martin G, Poot M, Nanda I, et al. GSE131752. Gene Expression Omnibus. 2019. https://www.ncbi.nlm.nih.gov/geo/query/acc.cgi?acc=GSE131752.

[80] Henderson AR, Wang Q, Meechoovet B, Siniard AL, Naymik M, De Both M, et al. DNA methylation and expression profiles of whole blood in Parkinson’s Disease. Front Genet. 2021;12:640266. 33981329 10.3389/fgene.2021.640266 PMC8107387

[81] Henderson A, Wang Q, Meechoovet B, Siniard A, Naymik M, DeBoth M, et al. GSE165081. Gene Expression Omnibus. 2021. https://www.ncbi.nlm.nih.gov/geo/query/acc.cgi?acc=GSE165081.

[82] Horvath S, Zhang Y, Langfelder P, Kahn RS, Boks MP, van Eijk K, et al. Aging effects on DNA methylation modules in human brain and blood tissue. Genome Biol. 2012;13(10): R97. 23034122 10.1186/gb-2012-13-10-r97 PMC4053733

[83] Horvath S, Zhang Y, Langfelder P, Kahn R, et al. GSE41169. Gene Expression Omnibus. 2012. https://www.ncbi.nlm.nih.gov/geo/query/acc.cgi?acc=GSE41169.

[84] Deary IJ, Gow AJ, Pattie A, Starr JM. Cohort profile: the Lothian Birth Cohorts of 1921 and 1936. Int J Epidemiol. 2012;41(6):1576–84. 22253310 10.1093/ije/dyr197

[85] Taylor AM, Pattie A, Deary IJ. Cohort profile update: the lothian birth cohorts of 1921 and 1936. Int J Epidemiol. 2018;47(4):1042-r. 29546429 10.1093/ije/dyy022 PMC6124629

[86] Xu Z, Niu L, Li L, Taylor J. ENmix: a novel background correction method for Illumina HumanMethylation450 BeadChip. Nucleic Acids Res. 2016;44(3):1–6. 26384415 10.1093/nar/gkv907 PMC4756845

[87] Xu Z, Langie SAS, De Boever P, Taylor JA, Niu L. RELIC: a novel dye-bias correction method for Illumina Methylation BeadChip. BMC Genomics. 2017;18(1):4. 28049437 10.1186/s12864-016-3426-3 PMC5209853

[88] Niu L, Xu Z, Taylor JA. RCP: a novel probe design bias correction method for Illumina Methylation BeadChip. Bioinformatics. 2016;32(17):2659–63. 27153672 10.1093/bioinformatics/btw285 PMC5013906

[89] Alisch RS, Barwick BG, Chopra P, Myrick LK, Satten GA, Conneely KN, et al. Age-associated DNA methylation in pediatric populations. Genome Res. 2012;22(4):623–32. 22300631 10.1101/gr.125187.111 PMC3317145

[90] Alisch R, Barwick B, Chopra P, Myrick L, et al. GSE36054. Gene Expression Omnibus. 2015; https://www.ncbi.nlm.nih.gov/geo/query/acc.cgi?acc=GSE36054.

[91] Harris R, Nagy-Szakal D, Pedersen N, Opekun A, et al. GSE32148. Gene expression omnibus. 2012. https://www.ncbi.nlm.nih.gov/geo/query/acc.cgi?acc=GSE32148.

[92] Harris RA, Nagy-Szakal D, Pedersen N, Opekun A, Bronsky J, Munkholm P, et al. Genome-wide peripheral blood leukocyte DNA methylation microarrays identified a single association with inflammatory bowel diseases. Inflamm Bowel Dis. 2012;18(12):2334–41. 22467598 10.1002/ibd.22956 PMC3812910

[93] Houtepen L, Vinkers C, Carrillo-Roa T, Hiemstra M, et al. GSE77445. Gene Expression Omnibus. 2016. https://www.ncbi.nlm.nih.gov/geo/query/acc.cgi?acc=GSE77445.

[94] Houtepen LC, Vinkers CH, Carrillo-Roa T, Hiemstra M, van Lier PA, Meeus W, et al. Genome-wide DNA methylation levels and altered cortisol stress reactivity following childhood trauma in humans. Nat Commun. 2016;7(1): 10967. 26997371 10.1038/ncomms10967 PMC4802173

[95] Gross A, Jaeger P, Kreisberg J, Licon K, Jepsen K, Khosroheidari M, et al. GSE67705. Gene Expression Omnibus. 2016. https://www.ncbi.nlm.nih.gov/geo/query/acc.cgi?acc=GSE67705.

[96] Hannum G, Guinney J, Zhao L, Zhang L, et al. GSE40279. Gene Expression Omnibus. 2012. https://www.ncbi.nlm.nih.gov/geo/query/acc.cgi?acc=GSE40279.

[97] Walker R, Liu J, Peters B, Ritz B, et al. GSE64495. Gene Expression Omnibus. 2015. https://www.ncbi.nlm.nih.gov/geo/query/acc.cgi?acc=GSE64495.

[98] Walker RF, Liu JS, Peters BA, Ritz BR, Wu T, Ophoff RA, et al. Epigenetic age analysis of children who seem to evade aging. Aging (Albany NY). 2015;7(5):334–9. 25991677 10.18632/aging.100744 PMC4468314

[99] Tan Q, Frost M, Heijmans BT, von Bornemann HJ, Tobi EW, Christensen K, et al. Epigenetic signature of birth weight discordance in adult twins. BMC Genomics. 2014;15(1):1062. 25476734 10.1186/1471-2164-15-1062 PMC4302120

[100] Tan Q, Christiansen L, Frost M. GSE61496. Gene Expression Omnibus. 2014. https://www.ncbi.nlm.nih.gov/geo/query/acc.cgi?acc=GSE61496.

[101] Arloth J, Binder E. GSE125105. Gene Expression Omnibus. 2019; https://www.ncbi.nlm.nih.gov/geo/query/acc.cgi?acc=GSE125105

[102] Arloth J, Eraslan G, Andlauer TFM, Martins J, Iurato S, Kühnel B, et al. DeepWAS: Multivariate genotype-phenotype associations by directly integrating regulatory information using deep learning. PLoS Comput Biol. 2020;16(2): e1007616. 32012148 10.1371/journal.pcbi.1007616 PMC7043350

[103] Liu Y, Aryee MJ, Padyukov L, Fallin MD, Hesselberg E, Runarsson A, et al. Epigenome-wide association data implicate DNA methylation as an intermediary of genetic risk in rheumatoid arthritis. Nat Biotechnol. 2013;31(2):142–7. 23334450 10.1038/nbt.2487 PMC3598632

[104] Liu Y, Feinberg A. GSE42861. Gene Expression Omnibus. 2013; https://www.ncbi.nlm.nih.gov/geo/query/acc.cgi?acc=GSE42861.

[105] Tsaprouni L, Yang T, Bell J, Dick K, Kanoni S, Nisbet J, et al. GSE50660. Gene Expression Omnibus. 2014. https://www.ncbi.nlm.nih.gov/geo/query/acc.cgi?acc=GSE50660.

[106] Tsaprouni LG, Yang TP, Bell J, Dick KJ, Kanoni S, Nisbet J, et al. Cigarette smoking reduces DNA methylation levels at multiple genomic loci but the effect is partially reversible upon cessation. Epigenetics. 2014;9(10):1382–96. 25424692 10.4161/15592294.2014.969637 PMC4623553

[107] Kular L, Liu Y, Ruhrmann S, Zheleznyakova G, Marabita F, Gomez-Cabrero D, et al. DNA methylation as a mediator of HLA-DRB1*15:01 and a protective variant in multiple sclerosis. Nat Commun. 2018;9(1):2397. 29921915 10.1038/s41467-018-04732-5 PMC6008330

[108] Liu Y. GSE106648. Gene Expression Omnibus. 2018. https://www.ncbi.nlm.nih.gov/geo/query/acc.cgi?acc=GSE106648.

[109] Perez-Correa JF, Stiehl T, Marioni RE, Corley J, Cox SR, Costa IG, et al. Weighted 2D-kernel density estimations provide a new probabilistic measure for epigenetic age - Additional files. Zenodo. 2025. 10.5281/zenodo.11489551. 10.1186/s13059-025-03562-1 PMC12016065 40264182

