# Supplementary material for: Weighted 2D-kernel density estimations provide a new probabilistic measure for epigenetic age
Source: Genome Biol. 2025 Apr 22;26:103. doi: 10.1186/s13059-025-03562-1 (PMC12016065; doi:10.1186/s13059-025-03562-1)
Supplement: Supplementary file 1 — Additional file 1. All supplemental figures and tables. Additional files 2-5. Available at https://doi.org/10.5281/zenodo.11489551 [109]. [file 13059_2025_3562_MOESM1_ESM.pdf]

Additional file 1

Weighted 2D-kernel density estimations provide a new probabilistic measure for epigenetic age

Juan-Felipe Perez-Correa, Thomas Stiehl, Riccardo E. Marioni, Janie Corley, Simon R. Cox, Ivan G. Costa, Wolfgang Wagner

Index

Supplemental Figures 2
- Figure S1. Kernel density maps of the 27 age-associated CpGs. 2
- Figure S2. Weighted approach with 491 CpG model. 3
- Figure S3. Multivariate linear models with different numbers of CpGs. 4
- Figure S4. Age predictions with the 27 CpG WKDE model in purified cell types. 5
- Figure S5. Nine CpG pyrosequencing-based WKDE clock. 6
- Figure S6. Mortality analysis in LBC1936. 7

Supplemental Tables 8
- Table S1. Datasets with methylation data for several diseases. 8
- Table S2. Mortality-associated CpGs of the 491 CpG signature in the LBC1921. 9
- Table S3. Mortality-associated CpGs of the 491 CpG signature in the LBC1936. 11
- Table S4. Mortality association of different covariates in LBC1921 and LBC1936. 13
- Table S5. Illumina BeadChip profiles used for training and validation sets. 14
- Table S6. List of 27 age-associated CpGs and variables for different models. 15
- Table S7. List of 491 age-associated CpGs and variables for different models. 16
- Table S8. Pyrosequencing data used for the 9CpG WKDE model. 26

Supplemental Figures

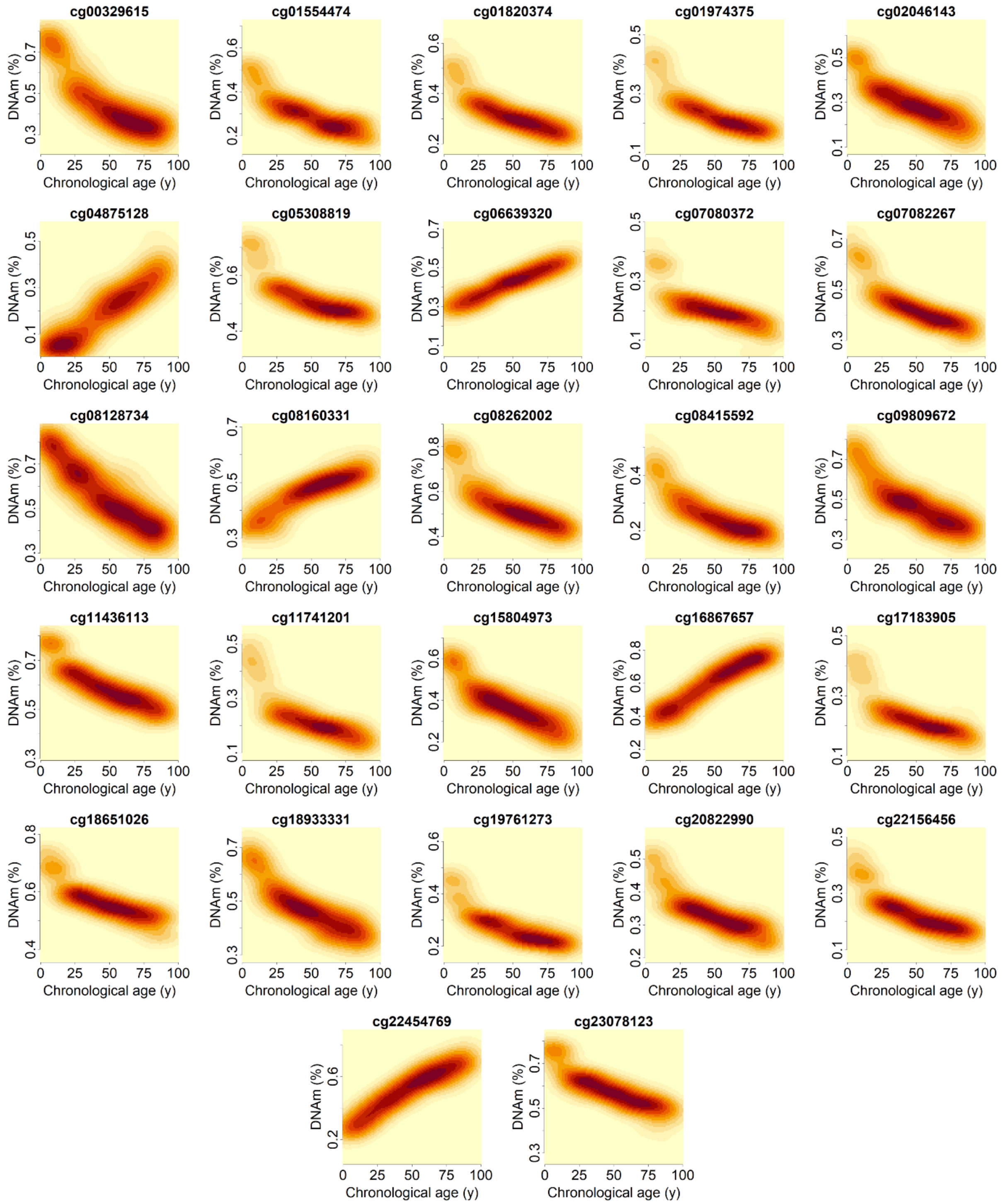

Figure S1. Kernel density maps of the 27 age-associated CpGs.

For the 27 age-associated CpGs the 2D density kernel estimations are depicted for a homogeneous age-distribution in the training set.

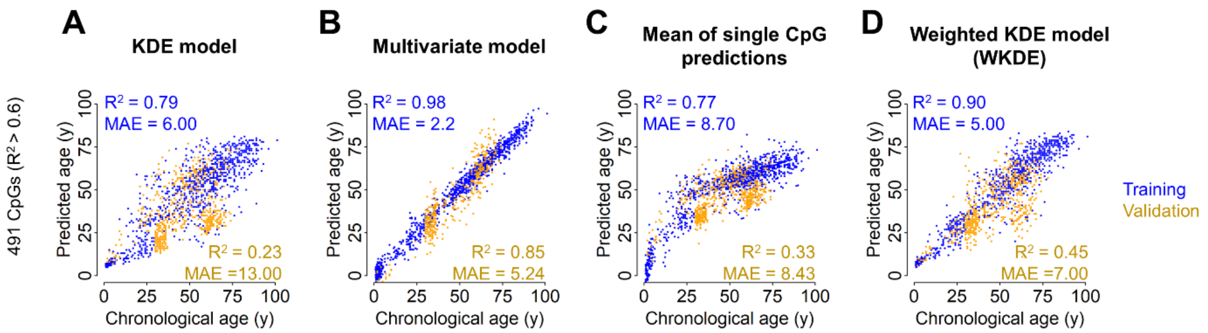

Figure S2. Weighted approach with 491 CpG model.

A) An alternative 2D kernel age predication model was generated for 491 CpGs ($R^2 > 0.6$ in the training set). The model was trained on a subset of samples from the training set with homogeneous age distribution. Pearson correlation R^2 and mean absolute error (MAE) are indicated either for all data of the training sets (blue; even though most of the samples were not selected for the homogeneous age presentation of the training data), and the independent validation sets (yellow). **B)** The same CpGs were used to generate multivariate models based on the entire training set. **C)** Alternatively, for each of the 491 age-associated CpGs a line regression model was established to facilitate single CpG predictions. The averaged of these predictions revealed a much lower precision of age predictions than for the multivariable model. **D)** The 491 CpG 2D kernel age-prediction model was further optimized by optimized weights for individual CpGs that were determined by genetic algorithm optimization. However, the performance in the validation set is lower than the one with the 27 CpG model.

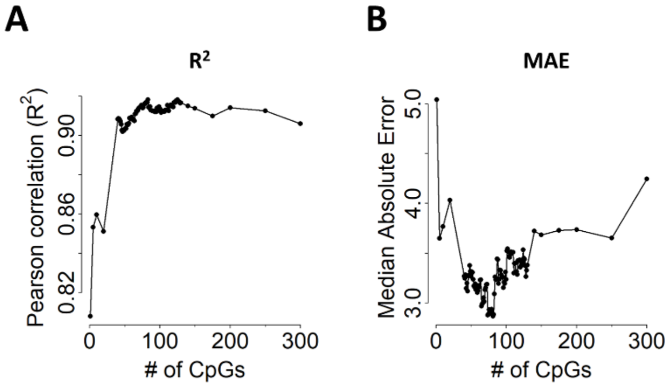

Figure S3. Multivariate linear models with different numbers of CpGs.

A) To test how the performance of multivariate linear models depends on the number of age-associated CpGs we trained such models with up to the top 300 age-associated CpGs in the training set. The highest Pearson correlation with chronological age was achieved with the model using 125 CpGs (R^2 = 0.92). **B)** Median absolute errors of the models mentioned in A. The smallest error was observed using the top 81 CpGs (MAE = 2.87 years).

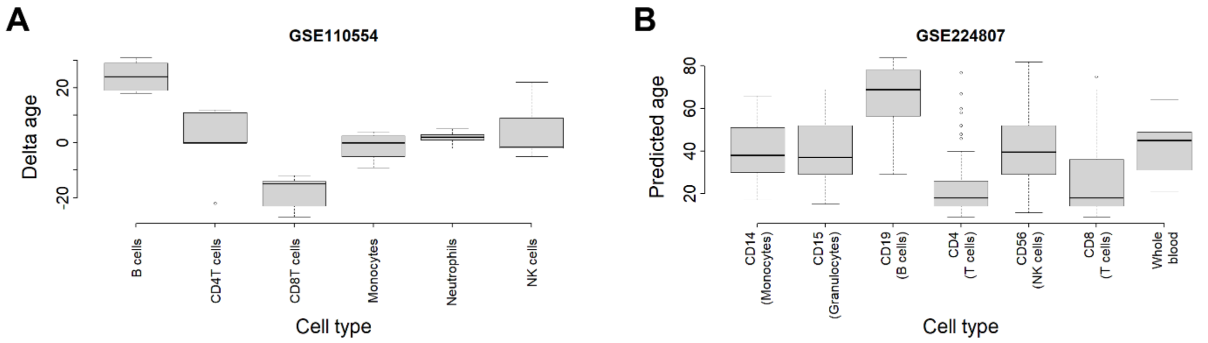

Figure S4. Age predictions with the 27 CpG WKDE model in purified cell types.

A) Delta age (predicted – chronological age) in different cell types of dataset GSE110554. CDT4 cells, monocytes, neutrophils and NK cell reveal age-predictions that are close to the corresponding chronological age (delta age close to 0 years). However, while CD8T cells reflect a decrease in age, B cells are marked by an increase in age. **B)** Predicted age in different cell types of dataset GSE224807. Monocytes, granulocytes and NK cells show similar predicted ages to whole blood measurements. In contrast, epigenetic age in purified B cells is predicted to be higher, whereas T cells are predicted younger.

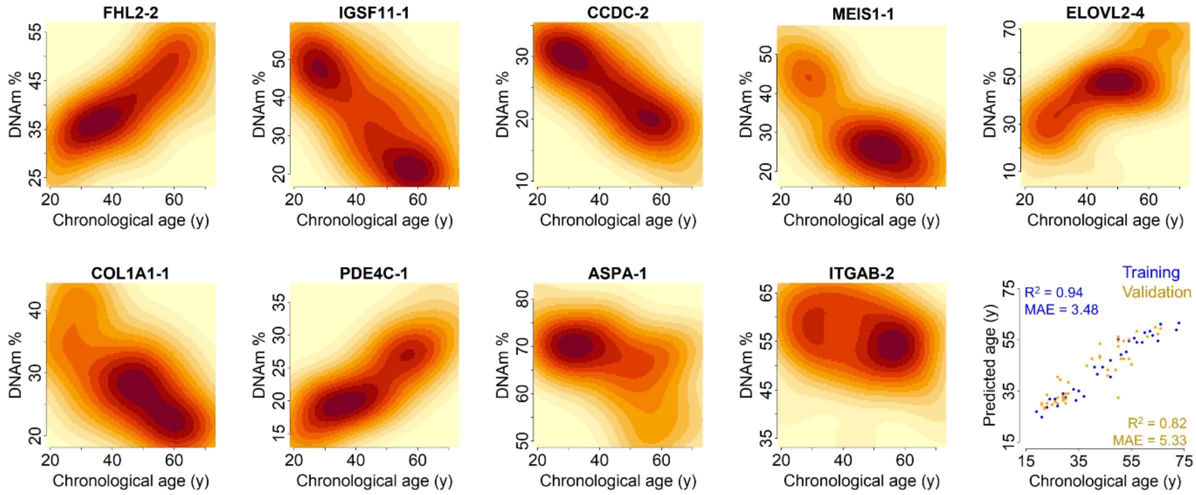

Figure S5. Nine CpG pyrosequencing-based WKDE clock.

Here we have exemplarily trained a WKDE model for pyrosequencing data to demonstrate applicability with targeted DNAm analysis. For each of the 9 age-associated CpGs the 2D density kernel estimations are depicted for a homogeneous age-distribution in the training set. The targeted WKDE clock reveals good association between the chronological and predicted age as well as a low median absolute error in both training (R^2 = 0.94, MAE = 3.48 years) and validation (R^2 = 0.82, MAE = 5.33 years) sets.

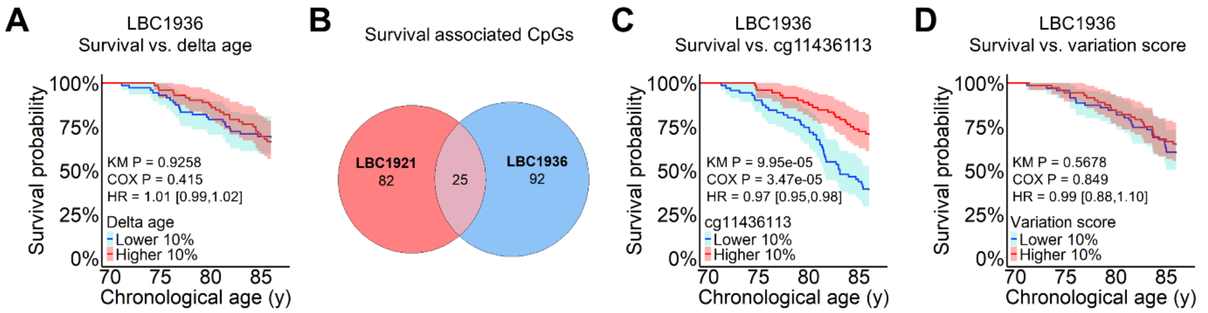

Figure S6. Mortality analysis in LBC1936.

A) The association of epigenetic age-predictions of the 27 WKDE model with all-cause mortality was also tested in the Lothian Birth Cohorts of 1936 (LBC1936). Kaplan-Meier survival curves for donors with highest and lowest 10% delta ages are depicted. Cox regression model for all donors, adjusted for chronological age and gender, showed no significant effect of delta age in mortality risk (HR = 1.005, 95% CI (0.9923, 1.0183), P < 0.415). **B)** Individual Cox regressions for all the 491 age-associated CpGs (adjusting for age and gender) revealed high overlap in mortality associated CpG sites in both cohorts. **C)** Kaplan-Meier survival curves for highest and lowest 10% methylation values of cg11436113 in LBC1936. Cox regression model, adjusted for chronological age and gender, shows that increase of 1% in the DNAm of cg11436113 is associated with a 3.30% decrease in mortality risk (95% CI (0.9520, 0.9830), P < 3.47E-5). **D)** Kaplan-Meier survival curves for highest and lowest 10% variation score in LBC1936. Cox regression model, adjusted for chronological age and gender, showed no significant effect of the variation score in mortality risk in the LBC1936 (HR = 0.9894, 95% CI (0.8870, 1.1037), P <0.849).

Supplemental Tables

Table S1. Datasets with methylation data for several diseases.

GEO Accession	Disease	Number of diseased	Number of healthy or control	Chronological age information	Tissue of samples
GSE124413	AML	459	41	No	Peripheral blood/Bone marrow
GSE133986	AML	64	0	Yes	Bone marrow
GSE153347	AML	105	0	No	Bone marrow
GSE62298	AML	68	0	No	Whole blood
GSE58477	AML	62	10	No	Bone marrow/Leukemic blast
GSE152710	AML	73	10	No	Bone marrow
GSE118241	Myelofibrosis	39	6	No	Peripheral blood/Bone marrow
GSE52588	Down syndrome	29	58	Yes	Whole blood
GSE67751	HIV	23	69	Yes	Whole blood
GSE131752	Progeroid syndrome	24	24	Yes	Whole blood
GSE165081	Parkinson's disease	14	14	No	Whole blood
GSE41169	Schizophrenia	62	33	Yes	Whole blood

Table S2. Mortality-associated CpGs of the 491 CpG signature in the LBC1921.

Site	HR	low CI 95%	up CI 95%	Pr(>|z|)	Slope (DNAm/age)	Gene	CHR
cg04875128	0,98	0,97	1,00	0,02	168,76	*OTUD7A*	15
cg08128734	0,99	0,98	1,00	0,00	-156,31	*RASSF5*	1
cg11436113	0,98	0,97	0,99	0,01	-234,05		20
cg19761273	0,97	0,95	1,00	0,04	-261,03	*CSNK1D*	17
cg19670290	0,97	0,96	0,98	0,00	-227,09	*HDDC3;UNC45A*	15
cg23003085	0,91	0,86	0,96	0,00	-228,96	*PRDX5;TRMT112*	11
cg23677833	0,89	0,84	0,95	0,00	-261,23	*AP1M1*	19
cg01812894	0,98	0,97	0,99	0,00	-203,72	*ALDH1A1*	9
cg23950157	0,94	0,91	0,97	0,00	-288,76	*COL1A1*	17
cg21990700	0,96	0,94	0,98	0,00	-207,22	*LOC283314;C1RL*	12
cg14934280	0,95	0,92	0,98	0,00	-285,25	*GRLF1*	19
cg06007201	0,91	0,86	0,96	0,00	-311,93	*FAM38A*	16
cg21635307	0,95	0,92	0,98	0,00	-268,86	*ANKRD6*	6
cg03206537	0,96	0,94	0,98	0,00	-199,68	*CTSA*	20
cg20813374	0,97	0,96	0,99	0,00	-253,22	*FKBP5*	6
cg27209729	0,98	0,97	0,99	0,00	-172,72	*NRXN2*	11
cg15393702	0,99	0,98	1,00	0,00	-191,53	*ANKRD29*	18
cg24155190	0,90	0,84	0,97	0,00	-303,80	*CSRP1*	1
cg17593342	0,98	0,97	1,00	0,00	-208,72		6
cg09608765	0,96	0,94	0,99	0,00	-266,54	*LIMD1*	3
cg08913523	0,96	0,94	0,99	0,00	-189,17		8
cg21922223	0,92	0,86	0,97	0,01	-343,10		17
cg26158023	0,96	0,93	0,99	0,01	-326,01	*CCBP2*	3
cg19453093	0,98	0,97	1,00	0,01	-197,53	*KCNK1*	14
cg26808293	0,94	0,90	0,98	0,01	-434,47	*TNFRSF12A*	16
cg01243823	0,97	0,96	0,99	0,01	-161,72	*NOD2*	16
cg00602811	0,98	0,97	1,00	0,01	-141,70	*ZEB2*	2
ch.1.171672612F	0,72	0,57	0,91	0,01	-347,67		1
cg13823169	0,98	0,97	1,00	0,01	-247,99		9
cg08301612	0,98	0,96	0,99	0,01	-175,99	*HEXIM1*	17
cg25994988	0,98	0,97	1,00	0,01	-237,33	*UBASH3B*	11
cg04080625	0,98	0,96	0,99	0,01	-268,41	*KIAA126*	1
cg18505959	0,93	0,87	0,98	0,01	-387,82	*BAT5*	6
cg06285727	0,94	0,90	0,99	0,01	-213,65	*ATG16L2*	11
cg21469505	0,98	0,97	1,00	0,01	-195,72		18
cg03881294	0,95	0,92	0,99	0,01	-216,31		2
cg04542977	0,98	0,97	1,00	0,01	-185,62	*EPS15*	1
cg25424279	0,97	0,96	0,99	0,01	-260,30		11
cg27386529	0,98	0,97	1,00	0,01	-227,37	*SCAP*	3
cg26101277	0,96	0,93	0,99	0,01	-202,69	*CCL1*	17
cg05316627	0,99	0,98	1,00	0,01	-185,21		6
cg25311470	0,98	0,96	0,99	0,01	-233,58	*NRCAM*	7
cg18797590	0,99	0,98	1,00	0,01	-191,60	*CC2D2A*	4
cg08471846	0,98	0,96	1,00	0,01	-267,28	*PTBP1*	19
cg14058848	0,98	0,96	1,00	0,01	-258,49	*TTC16;PTRH1*	9
cg12197142	0,94	0,90	0,99	0,01	-315,66	*RECQL5;LOC6438*	17
ch.2.47286786F	0,34	0,15	0,81	0,01	-448,44		2
cg19784428	0,99	0,98	1,00	0,02	-185,42	*NWD1*	19
cg04474832	0,97	0,95	0,99	0,02	-318,26	*ABHD14B;ABHD14A*	3
cg05379350	0,97	0,94	0,99	0,02	-257,55	*GIT1*	17

cg02610723	0,96	0,92	0,99	0,02	-330,23	*FAM38A*	16
cg03746976	0,98	0,96	1,00	0,02	-228,85	*C16orf57*	16
cg23836737	0,98	0,97	1,00	0,02	-237,23		4
cg16677191	0,97	0,94	0,99	0,02	-191,76	*GLRX*	5
cg27470213	0,96	0,93	0,99	0,02	-264,14	*LGALS3BP*	17
cg12483947	0,97	0,95	1,00	0,02	-208,51	*SGPL1*	10
cg13072940	0,98	0,96	1,00	0,02	-256,79	*MON1A*	3
cg01955153	0,96	0,93	0,99	0,02	-271,17		16
cg19729744	0,99	0,98	1,00	0,02	-151,66		3
cg26894354	0,98	0,97	1,00	0,02	-228,87	*FMOD*	1
cg16363586	0,95	0,92	0,99	0,03	-241,65	*BST2*	19
cg14314729	0,98	0,97	1,00	0,03	-225,50		5
cg07027613	0,96	0,92	1,00	0,03	-197,46	*C1RL;LOC283314*	12
ch.13.39564907R	0,74	0,56	0,97	0,03	-308,24		13
cg01511567	0,97	0,94	1,00	0,03	-267,54	*SSRP1*	11
cg18779283	0,93	0,87	0,99	0,03	-365,20	*LIMD1*	3
cg06647068	0,98	0,97	1,00	0,04	-197,14	*CHST11*	12
cg08090640	0,99	0,97	1,00	0,04	-219,26	*IFI35*	17
cg22864266	0,96	0,93	1,00	0,04	-354,17	*FKBP11*	12
cg13428009	0,86	0,75	0,99	0,04	-371,17		9
cg05242244	0,98	0,97	1,00	0,04	-320,59	*CSAD*	12
cg19936954	0,98	0,95	1,00	0,04	-259,98	*QSOX1*	1
cg27004870	0,96	0,93	1,00	0,04	-328,68	*FAM38A*	16
cg16618104	0,96	0,93	1,00	0,04	-252,79	*CHST11*	12
cg26954174	0,97	0,95	1,00	0,04	-168,23	*NOD2*	16
cg22976533	0,99	0,98	1,00	0,04	-189,14	*PACS2*	14
cg08234504	0,97	0,95	1,00	0,04	-279,76		5
cg20595453	0,99	0,97	1,00	0,04	-227,30	*VPS52*	6
cg03725309	0,96	0,92	1,00	0,04	-175,69	*SARS*	1
cg20747538	0,98	0,96	1,00	0,04	-247,03		3
cg12179661	0,99	0,97	1,00	0,05	-269,98	*ENTPD8*	9
cg12009872	0,97	0,95	1,00	0,05	-269,47	*CYP19A1*	15

The first 4 CpG sites are also mortality associated in the list of 27CpGs (with R2 > 0.7 between DNAm and age in the training set).

CpG sites also significantly associated with all-cause mortality in the meta-EWAS from Bernabeu et. al. study

CpG sites significantly associated with all-cause mortality in the meta-EWAS from Bernabeu et. al. study AND in the LBC1936

Table S3. Mortality-associated CpGs of the 491 CpG signature in the LBC1936.

Site	HR	low CI 95%	up CI 95%	Pr(>|z|)	Slope (DNAm/age)	Gene	CHR
cg11436113	0,97	0,95	0,98	0,00	-234,05		20
cg01594949	0,97	0,96	0,99	0,00	-239,86	*NRARP*	9
cg05405914	0,97	0,96	0,99	0,00	-228,23		16
cg14775286	0,97	0,96	0,99	0,00	-203,10	*DZIP1L*	3
cg14989226	0,97	0,95	0,99	0,00	-219,92	*SRRM3*	7
cg19578183	0,96	0,94	0,98	0,00	-265,46		2
cg20813374	0,97	0,95	0,98	0,00	-253,22	*FKBP5*	6
cg01243823	0,97	0,96	0,99	0,00	-161,72	*NOD2*	16
cg06647068	0,98	0,96	0,99	0,00	-197,14	*CHST11*	12
cg08471846	0,96	0,94	0,99	0,00	-267,28	*PTBP1*	19
cg16541026	0,97	0,96	0,99	0,00	-236,27	*P4HTM*	3
cg16664617	0,96	0,94	0,98	0,00	-293,24	*SLC27A1*	19
cg17436656	0,98	0,96	0,99	0,00	-231,24	*RARG*	12
cg20595453	0,97	0,95	0,99	0,00	-227,30	*VPS52*	6
cg24848615	0,97	0,96	0,99	0,00	-220,80	*NFIC*	19
cg08713098	0,96	0,94	0,99	0,00	-264,02	*ZCWPW1*	7
cg20988565	0,98	0,97	0,99	0,00	-171,10	*ZFPM2*	8
cg23003085	0,92	0,88	0,97	0,00	-228,96	*PRDX5;TRMT112*	11
cg23677833	0,90	0,85	0,96	0,00	-261,23	*AP1M1*	19
cg11331344	0,97	0,94	0,99	0,00	-288,68	*RECQL5;LOC6438*	17
cg13501527	0,98	0,96	0,99	0,00	-292,84	*PHF19*	9
cg13823169	0,98	0,96	0,99	0,00	-247,99		9
cg20912205	0,95	0,93	0,98	0,00	-265,50	*NAT6;HYAL3*	3
cg21990700	0,97	0,94	0,99	0,00	-207,22	*LOC283314;C1RL*	12
cg22156842	0,97	0,96	0,99	0,00	-172,15	*TMEM22*	3
cg05316627	0,99	0,97	1,00	0,00	-185,21		6
cg14188401	0,96	0,94	0,99	0,00	-296,18		3
cg19453093	0,98	0,97	0,99	0,00	-197,53	*KCNK1*	14
cg01234420	0,98	0,97	1,00	0,01	-154,57	*LOC15381*	22
cg02402091	0,98	0,96	0,99	0,01	-218,54	*ACSL5*	10
cg04581938	0,83	0,73	0,95	0,01	-335,93		6
cg04959790	0,98	0,96	0,99	0,01	-234,97	*NR1H3*	11
cg05237436	0,98	0,96	0,99	0,01	-157,19	*SIL1*	5
cg19261426	0,98	0,97	1,00	0,01	-209,97	*OBSCN*	1
cg21139312	1,11	1,03	1,20	0,01	300,34	*MSI2*	17
cg06240854	0,98	0,97	1,00	0,01	-217,23	*LIPT1;TSGA1*	2
cg08468689	0,97	0,95	0,99	0,01	-254,36	*GHDC*	17
cg24711336	0,98	0,96	0,99	0,01	-254,99		10
cg26954174	0,97	0,95	0,99	0,01	-168,23	*NOD2*	16
ch.1.839062R	0,87	0,78	0,97	0,01	-333,21	*RUNX3*	1
cg12688670	0,97	0,94	0,99	0,01	-322,50	*KIF22*	16
cg13066481	0,98	0,96	0,99	0,01	-228,44	*MYLK*	3
cg26166595	0,98	0,96	1,00	0,01	-211,39	*DKK3*	11
cg00863306	0,91	0,84	0,98	0,01	-301,57	*NANOS3*	19
cg04424621	0,94	0,89	0,99	0,01	-249,37	*HIST1H2BJ*	6
cg18993949	0,98	0,96	1,00	0,01	-263,76	*CC2D2A*	4
cg21572722	1,03	1,01	1,05	0,01	333,99	*ELOVL2*	6
cg06142740	0,98	0,96	1,00	0,01	-300,47	*PPP1CB*	2
cg07368443	0,98	0,96	1,00	0,01	-295,40	*PLK3*	1
cg13807549	0,98	0,96	1,00	0,01	-220,42		9

cg05045027	0,98	0,97	1,00	0,01	-226,11		11
cg13428009	0,85	0,75	0,97	0,01	-371,17		9
cg15416179	0,80	0,68	0,96	0,01	-310,10	*MAP2K3*	17
cg26276120	0,96	0,93	0,99	0,01	-312,80	*TPI1*	12
cg25371036	0,98	0,96	1,00	0,02	-231,10	*AMOTL1*	11
cg16363586	0,95	0,91	0,99	0,02	-241,65	*BST2*	19
cg19344626	0,99	0,97	1,00	0,02	-160,82	*NWD1*	19
cg20964856	0,98	0,96	1,00	0,02	-271,65	*HNRNPUL1;AXL*	19
cg08913523	0,97	0,94	0,99	0,02	-189,17		8
cg02010481	0,97	0,94	0,99	0,02	-158,13	*JAZF1*	7
cg06661266	0,99	0,98	1,00	0,02	-200,46		3
cg11807280	0,99	0,98	1,00	0,02	-120,85		2
cg18786171	0,98	0,97	1,00	0,02	-248,96	*ADK*	10
ch.1.171672612F	0,77	0,62	0,96	0,02	-347,67		1
cg02610723	0,96	0,93	1,00	0,03	-330,23	*FAM38A*	16
cg03206537	0,97	0,94	1,00	0,03	-199,68	*CTSA*	20
cg16677191	0,97	0,94	1,00	0,03	-191,76	*GLRX*	5
cg18215449	0,99	0,98	1,00	0,03	-177,35		12
cg09124496	0,99	0,98	1,00	0,03	-176,00	*LOC285954;INHBA*	7
cg24847230	0,98	0,96	1,00	0,03	-345,09	*UBE2Z*	17
cg05619598	0,98	0,97	1,00	0,03	-197,92		19
cg06285727	0,95	0,91	1,00	0,03	-213,65	*ATG16L2*	11
cg26969888	0,98	0,96	1,00	0,03	-201,74	*PODNL1;DCAF15*	19
ch.6.33611621F	0,63	0,42	0,96	0,03	-328,25		6
cg00103778	0,98	0,96	1,00	0,03	-217,65		20
cg14977938	1,02	1,00	1,05	0,03	-258,46	*ZFYVE21*	14
cg07843120	0,93	0,88	0,99	0,03	-264,09	*GPI*	19
ch.15.67797584R	0,92	0,84	0,99	0,04	-236,06		15
cg19056004	0,99	0,97	1,00	0,04	-227,46	*LRRC23;ENO2*	12
cg26543112	0,97	0,95	1,00	0,04	-234,51		6
cg08957484	0,99	0,97	1,00	0,04	198,18	*CCNI2*	5
cg16810343	0,98	0,97	1,00	0,04	-244,57	*SRRM3*	7
cg00753885	0,97	0,94	1,00	0,04	-352,74		12
cg08301612	0,98	0,96	1,00	0,04	-175,99	*HEXIM1*	17
cg01981760	0,95	0,90	1,00	0,04	-423,95	*FTO;RPGRIP1L*	16
cg19784428	0,99	0,97	1,00	0,04	-185,42	*NWD1*	19
cg23744638	0,99	0,98	1,00	0,04	-177,22		11
cg12179661	0,98	0,97	1,00	0,05	-269,98	*ENTPD8*	9
cg15034393	0,98	0,97	1,00	0,05	-150,56		3
cg25424279	0,98	0,96	1,00	0,05	-260,30		11
cg02797271	0,93	0,86	1,00	0,05	-311,15	*GPR132*	14
cg26210267	0,97	0,94	1,00	0,05	-303,00	*ATP5I*	4

The first CpG site is also mortality associated in the list of 27CpGs (with R2 > 0.7 between DNAm and age in the training set).

[orange] CpG sites significantly associated with all-cause mortality in the meta-EWAS from Bernabeu et. al. study

[blue] CpG sites significantly associated with all-cause mortality in the meta-EWAS from Bernabeu et. al. study AND in the LBC1921

Table S4. Mortality association of different covariates in LBC1921 and LBC1936.

Covariate	LBC1921				LBC1936			
	**HR**	**95% CI lower**	**95% CI upper**	**P**	**HR**	**95% CI lower**	**95% CI upper**	**P**
Age	0,82	0,64	1,05	0,1229	0,88	0,72	1,07	0,2043
Sex	0,54	0,33	0,88	**0,0140**	0,40	0,23	0,69	**0,0011**
Delta age	0,99	0,97	1,01	0,2751	0,99	0,98	1,01	0,4551
Variation score	0,86	0,78	0,96	**0,0075**	1,01	0,88	1,15	0,9407
Smoker	0,96	0,77	1,20	0,6960	1,05	1,03	1,08	**0,0000**
Basophil	1,04	1,00	1,08	**0,0477**	1,01	0,95	1,08	0,6376
Eosinophil	1,00	0,97	1,02	0,7922	1,00	0,95	1,05	0,9532
Monocyte	1,00	0,97	1,02	0,7457	1,00	0,95	1,05	0,9591
Lymphocite	1,00	0,98	1,02	0,9739	1,00	0,96	1,05	0,9043
Neutrophil	1,00	0,98	1,02	0,9473	1,00	0,96	1,05	0,8639
White cell count	1,00	0,98	1,02	0,8911	1,00	0,95	1,05	0,8779
Platelet	1,00	1,00	1,00	0,2364	1,00	1,00	1,00	0,0677
Triglyceride	0,94	0,79	1,13	0,5236	1,04	0,84	1,27	0,7444
Cholesterol	0,88	0,76	1,02	0,0897	0,90	0,78	1,04	0,1458
Heart rate	0,99	0,98	1,00	0,1578	-	-	-	-
BMI	0,98	0,95	1,02	0,3719	1,03	0,99	1,06	0,1083
Alcohol consumption	1,01	0,99	1,03	0,2442	1,01	1,00	1,02	0,0884
Sitting diastolic pressure	1,01	1,00	1,02	0,1831	1,00	0,99	1,01	0,9045
Forced expiratory volume	0,71	0,51	1,00	**0,0495**	0,66	0,50	0,89	**0,0059**
Grip strength	0,99	0,96	1,01	0,3361	0,99	0,96	1,01	0,2637
Vitamin B12	1,00	1,00	1,00	0,3702	1,00	1,00	1,00	0,2369
Telomere length	1,00	1,00	1,00	0,2411	1,00	1,00	1,00	0,9215
Activity score*	0,95	0,93	0,98	**0,0007**	1,01	0,87	1,17	0,8788
Life quality	1,35	1,03	1,77	**0,0322**	0,77	0,60	0,99	**0,0427**
Health satisfaction	0,79	0,65	0,96	**0,0164**	0,89	0,74	1,08	0,2315
Life enjoyment	1,02	0,78	1,33	0,9073	0,95	0,71	1,27	0,7236

The significant covariates (P <= 0.05) are highlighted in bold.
*Activity lifestyle score in LBC1921, Physical activity level in LBC1936

Table S5. Illumina BeadChip profiles used for training and validation sets.

GEO Accession	Number of samples	Number of healthy or control	Age range healthy or control	Gender distribution (male%)	Tissue of samples	Set
GSE36054	192	134	1-17 y	58,96%	Leukocytes	Training
GSE32148	48	19	3-76 y	36,84%	Periph. blood	Training
GSE41169	95	33	18-65 y	63,64%	Whole blood	Training
GSE77445	85	85	18-69 y	50,59%	Whole blood	Training
GSE52588	87	58	9-83 y	28,74%	Whole blood	Training
GSE67705	284	44	27-66 y	65,49%	Whole blood	Training
GSE40279	656	656	19-101 y	48,48%	Whole blood	Training
**Total Training**	**1447**	**1029**	**1-101 y**	**49,90%**		
GSE64495	113	106	2-73 y	32,74%	Whole blood	Validation
GSE61496	312	312	30-74 y	52,56%	Whole blood	Validation
GSE125105	699	210	19-79 y	44,64%	Whole blood	Validation
GSE42861	689	101	24-70 y	21,78%	Periph. blood leuk.	Validation
GSE50660	464	179	40-65 y	67,60%	Periph. blood	Validation
GSE106648	279	72	20-65 y	27,78%	Periph. blood	Validation
**Total Validation**	**2556**	**980**	**2-79 y**	**46,47%**		

Table S6. List of 27 age-associated CpGs and variables for different models.

CpG	Coefficients of multivariate models	Slope of individual linear regression	Intercept of individual linear regression	Weights of WKDE	CHR	LOC	Strand	UCSC Ref. Gene Name
(Intercept)	34,77	-	-	-				
cg00329615	-11,01	-145,07	118,47	2,21	3	118706648	-	*CASZ1*
cg01554474	-13,42	-214,62	117,38	1,73	1	155107599	+	*DGKI*
cg01820374	8,61	-223,49	123,03	0,72	12	6882083	-	
cg01974375	21,81	-250,97	112,92	-0,82	1	151298954	+	
cg02046143	-5,03	-193,35	106,50	6,36	11	133797911	+	*SMAP1*
cg04875128	31,77	168,76	10,48	6,95	15	31775895	-	*HDAC4*
cg05308819	-38,35	-241,64	178,70	1,96	1	155959156	-	*HINT2*
cg06639320	86,65	252,91	-59,85	6,41	2	106015739	-	
cg07080372	-47,81	-267,00	107,14	3,38	11	796607	-	*IGSF11*
cg07082267	13,11	-209,06	142,41	2,03	16	85429035	-	*CASS4*
cg08128734	-13,57	-156,31	138,19	2,34	1	206685423	-	*PTGDS*
cg08160331	4,02	274,45	-76,25	2,45	11	75140865	+	
cg08262002	9,84	-184,95	151,48	1,03	4	16575323	+	*ZEB2*
cg08415592	-18,56	-228,55	113,12	1,85	22	36648973	-	*ZEB2*
cg09809672	-13,55	-163,69	131,16	3,69	1	236557682	-	*TCEA2*
cg11436113	-13,00	-234,05	189,57	3,47	20	19191145	-	
cg11741201	3,38	-210,59	102,49	-0,65	11	35638398	-	*TJP2*
cg15804973	-3,36	-177,55	118,91	1,29	6	137114513	+	*MMEL1*
cg16867657	55,03	173,46	-55,16	7,63	6	11044877	+	
cg17183905	-18,78	-242,45	107,33	1,26	12	110253842	-	
cg18651026	14,14	-270,92	203,20	0,28	6	33140660	-	*DDO*
cg18933331	-17,34	-210,85	151,07	1,42	1	110186418	-	*NANOS3*
cg19761273	20,50	-261,03	123,42	-3,47	17	80232096	+	*CD300LB*
cg20822990	9,64	-270,87	143,67	-2,01	1	17338766	-	
cg22156456	26,88	-247,48	108,72	-0,42	17	39844239	-	*SNX20*
cg22454769	-24,50	149,85	-29,55	3,79	2	106015767	-	*MIR770;MEG3*
cg23078123	-22,03	-232,69	187,66	-0,05	1	68577796	+	*EHD2*

For each of the 27 CpGs, we include the coefficients for the multivariate model, the slopes and intercepts for the average of n-independent linear regressions, and the weights for the WKDE model.

Table S7. List of 491 age-associated CpGs and variables for different models.

CpG	Coefficients of multivariate model	Slopes of individual regression	Intercepts of indiv. reg.	Weight of WKDE model	CHR	LOC	Strand	UCSC Ref. Gene Name
(Intercept)	21,88							
cg00003345	0,02	-294,32	231,35	0,70	1	10816499	-	*CASZ1*
cg00029246	14,34	-149,13	121,16	1,07	7	137223417	+	*DGKI*
cg00101260	6,52	-247,53	122,71	1,20	17	53426657	-	
cg00103778	3,83	-217,65	112,68	1,59	20	30196041	+	
cg00129827	0,76	-237,60	106,88	0,26	6	71394664	+	*SMAP1*
cg00144180	32,77	218,95	-134,16	2,16	2	240294362	-	*HDAC4*
cg00193668	3,51	-255,58	188,14	-0,87	9	35814283	+	*HINT2*
cg00232105	-1,66	-274,93	211,60	5,79	14	100752338	+	
cg00329615	1,51	-145,07	118,47	2,39	3	118706648	-	*IGSF11*
cg00387658	-10,49	-236,71	182,05	-1,25	20	54986793	+	*CASS4*
cg00563932	-6,61	-218,23	158,22	5,68	9	139871049	+	*PTGDS*
cg00570618	8,16	-211,86	84,53	0,04	8	81143035	+	
cg00573770	-0,18	-133,82	104,46	2,31	2	145278485	+	*ZEB2*
cg00602811	-7,13	-141,70	113,11	3,29	2	145278564	+	*ZEB2*
cg00636737	0,06	-287,28	259,41	-2,85	20	62695569	-	*TCEA2*
cg00655552	7,28	-209,87	73,30	-3,56	4	182862370	-	
cg00695112	16,13	-269,01	222,26	0,52	9	71862747	-	*TJP2*
cg00695391	4,73	-177,87	118,04	-2,20	1	2525548	-	*MMEL1*
cg00753885	-8,64	-352,74	310,14	3,56	12	57401797	-	
cg00792107	-7,15	-223,29	119,29	1,35	12	133181600	-	
cg00804078	3,24	-124,53	96,74	-1,73	6	110736941	+	*DDO*
cg00863306	-1,53	-301,57	96,15	-6,56	19	13991470	+	*NANOS3*
cg00873351	11,21	-193,91	125,32	-0,24	17	72527813	+	*CD300LB*
cg00876267	12,39	-256,34	210,19	3,69	9	139588516	+	
cg00921350	1,33	-253,07	194,05	-3,64	16	50701499	-	*SNX20*
cg01022345	-19,99	-292,83	232,39	-7,15	14	101317819	+	*MIR770;ME*
cg01055871	1,74	-291,65	120,98	0,41	19	48216390	+	*EHD2*
cg01102833	-3,60	-253,36	115,79	-2,00	4	176986621	-	*WDR17*
cg01102854	-16,03	-234,09	174,69	1,36	11	63605717	+	*MARK2*
cg01214061	-5,06	-295,45	79,75	0,78	4	174290520	-	
cg01234420	9,90	-154,57	124,71	1,98	22	46453808	+	*LOC150381*
cg01243823	7,40	-161,72	103,98	-1,00	16	50732212	-	*NOD2*
cg01282174	5,98	-130,52	84,37	-1,20	11	119630144	+	
cg01314044	2,75	-206,84	110,33	2,60	11	94246036	+	
cg01447660	-13,11	-236,84	120,25	-1,74	2	69152678	-	
cg01502244	-26,15	-361,72	317,41	5,42	17	78188904	+	*SGSH*
cg01506917	3,23	-181,39	130,47	-2,34	6	42417086	-	*TRERF1*
cg01511567	1,83	-267,54	109,63	4,67	11	57103631	+	*SSRP1*
cg01514353	12,84	-227,11	118,43	5,02	22	46259643	-	
cg01528542	-14,06	-218,48	185,24	5,69	12	81468232	+	
cg01538166	25,68	-183,54	99,01	1,42	17	17743987	+	
cg01542019	-6,78	-162,25	137,60	0,57	19	14673053	-	*TECR*
cg01554474	-14,00	-214,62	117,38	2,25	1	155107599	+	*RAG1AP1*
cg01594949	5,88	-239,86	190,51	-2,28	9	140197890	+	*NRARP*
cg01649334	-10,00	-236,58	97,39	3,38	3	101547296	+	*NFKBIZ*
cg01676322	21,15	-443,73	117,39	-0,74	17	43213429	-	*ACBD4*
cg01719405	-12,52	-169,81	120,33	4,37	14	62401258	+	
cg01812894	-0,92	-203,72	167,12	8,88	9	75568506	-	*ALDH1A1*
cg01820374	1,36	-223,49	123,03	0,58	12	6882083	-	*LAG3*

cg01881062	-5,41	-202,29	119,73	-0,61	1	6660403	+	*KLHL21*
cg01910639	-24,17	-254,33	207,95	1,92	1	153507779	-	*S100A6*
cg01955153	17,37	-271,17	101,76	-6,88	16	50769852	+	
cg01974375	-8,76	-250,97	112,92	0,84	1	151298954	+	*PI4KB*
cg01981760	-2,86	-423,95	117,46	-1,72	16	53737576	+	*FTO*
cg02010481	3,25	-158,13	85,54	-4,57	7	28218524	-	*JAZF1*
cg02030542	-8,10	-223,01	174,64	6,60	16	70722454	+	*VAC14*
cg02046143	4,31	-193,35	106,50	2,86	11	133797911	+	*IGSF9B*
cg02151301	7,17	-276,14	146,28	8,57	20	30101785	-	*HM13*
cg02286081	-2,28	-180,56	86,08	0,41	6	33043841	-	*HLA-DPB1*
cg02361903	-7,32	-214,84	145,95	-0,40	1	110452616	-	*CSF1*
cg02395812	5,16	-332,13	159,08	-2,60	14	105955879	+	*C14orf80*
cg02402091	-5,10	-218,54	150,84	0,52	10	114135092	+	*ACSL5*
cg02610723	-37,22	-330,23	97,50	7,64	16	88850534	-	*FAM38A*
cg02797271	18,65	-311,15	95,41	1,56	14	105532159	+	*GPR132*
cg03039990	15,05	-193,86	126,97	0,80	14	101317622	-	*MIR770*
cg03043157	-3,54	-136,40	94,00	1,93	6	97010903	+	*FHL5*
cg03055693	5,14	-282,61	235,64	-5,86	20	60910388	-	*LAMA5*
cg03172991	36,91	-325,71	216,59	1,72	19	13105728	+	*NFIX*
cg03206537	6,34	-199,68	100,61	4,34	20	44521739	+	*CTSA*
cg03211864	8,94	-271,93	127,34	-0,74	10	124060833	+	*BTBD16*
cg03359362	4,68	-338,44	84,27	-1,04	19	47289611	-	*SLC1A5*
cg03431918	4,33	-236,94	94,41	-3,99	17	77716367	-	
cg03443986	-15,08	-308,88	94,33	1,59	2	65100572	+	
cg03474926	32,86	-340,34	181,38	1,06	9	136023407	-	*RALGDS*
cg03530962	-3,72	-239,81	202,14	2,16	3	58475833	-	
cg03551243	2,08	-389,39	92,04	-1,37	19	5718912	-	*LONP1*
cg03638795	-5,06	-254,32	114,77	-9,20	11	416499	+	*SIGIRR*
cg03643998	18,69	-293,15	90,68	0,13	17	77042455	-	*C1QTNF1*
cg03698343	-12,00	-315,12	187,58	4,52	9	139921892	+	*ABCA2*
cg03725309	-16,54	-175,69	87,92	-0,17	1	109757585	+	*SARS*
cg03735592	1,66	-245,01	213,08	6,99	6	138821354	-	*NHSL1*
cg03746976	-19,66	-228,85	147,67	2,32	16	58035805	-	*C16orf57*
cg03881294	28,20	-216,31	88,92	1,39	2	11884333	-	
cg03922748	11,92	-241,06	79,62	2,09	2	220142903	+	*DNAJB2*
cg03982897	-20,37	-292,90	198,44	-3,57	11	1990108	+	
cg04080625	7,46	-268,41	146,41	-4,91	1	15426634	+	*KIAA1026*
cg04100595	5,63	-150,16	75,64	5,24	12	27615138	-	
cg04193015	4,93	-207,20	176,67	-2,65	17	35596680	+	*ACACA*
cg04200607	3,66	-207,63	107,88	-2,85	10	72163475	+	*EIF4EBP2*
cg04208403	3,20	-318,53	236,09	5,06	16	49525807	+	*ZNF423*
cg04308040	10,66	-161,83	134,29	0,73	13	44942303	+	
cg04424621	-6,24	-249,37	96,01	2,18	6	27101941	+	*HIST1H2BJ*
cg04425624	3,47	-238,75	101,36	1,50	6	31543565	-	*TNF*
cg04436528	23,19	-313,55	237,13	-0,90	8	143554875	+	*BAI1*
cg04474832	1,87	-318,26	142,24	-1,35	3	52008487	+	*ABHD14B*
cg04542977	14,58	-185,62	106,88	3,86	1	51982669	+	*EPS15*
cg04561237	9,95	-247,07	87,52	-3,91	10	126430405	+	*FAM53B*
cg04581938	-16,62	-335,93	86,89	1,68	6	31364999	-	
cg04596060	-0,70	-309,79	86,81	8,04	12	114404600	+	*RBM19*
cg04604946	-16,82	-279,17	153,34	4,31	12	7023352	+	*LRRC23*
cg04651240	-5,86	-238,23	162,72	1,17	11	18229519	-	*LOC494141*
cg04666029	-10,46	-419,15	101,85	-1,58	11	2552843	-	*KCNQ1*
cg04819580	1,87	-255,71	106,45	-3,16	7	150635526	+	

cg04872610	17,99	-303,70	140,74	-8,12	1	114448489	-	*AP4B1*
cg04875128	14,79	168,76	10,48	0,81	15	31775895	-	*OTUD7A*
cg04890576	3,71	-147,88	108,77	1,23	17	73032613	+	
cg04956949	20,07	-258,85	191,77	1,58	7	73119262	+	*STX1A*
cg04959790	8,63	-234,97	182,52	3,92	11	47278977	+	*NR1H3*
cg04999352	1,72	-272,81	109,02	4,92	11	63304614	-	*RARRES3*
cg05045027	1,83	-226,11	133,14	-3,31	11	119331282	+	
cg05061804	-15,69	-287,78	118,27	-2,66	9	135282417	+	*TTF1*
cg05091997	4,87	-196,03	80,75	-0,77	17	60897721	+	
cg05156137	-3,56	-154,73	100,47	-0,97	21	35898975	-	*RCAN1*
cg05191655	4,41	-238,15	109,75	0,92	4	38162793	-	
cg05207048	-5,78	-147,46	79,61	0,62	5	167513456	-	*ODZ2*
cg05237436	-0,80	-157,19	103,98	-1,64	5	138533428	+	*SIL1*
cg05242244	-6,57	-320,59	183,73	4,01	12	53553086	-	*CSAD*
cg05308819	-3,67	-241,64	178,70	-6,06	1	155959156	-	
cg05316627	7,55	-185,21	154,67	-0,30	6	87861261	-	
cg05324516	-4,65	-252,03	232,93	2,79	10	29421357	-	
cg05369942	2,82	-264,76	102,42	-4,55	4	142228617	-	
cg05379350	-9,52	-257,55	114,82	-1,98	17	27917157	-	*GIT1*
cg05405914	-1,95	-228,23	172,28	0,78	16	8972233	+	
cg05412028	-2,14	-125,85	67,37	-2,16	13	95952937	-	*ABCC4*
cg05584950	3,76	-212,69	93,92	-2,25	2	86012847	-	*ATOH8*
cg05619598	-6,09	-197,92	136,07	-0,99	19	33569228	-	
cg05694021	1,07	-119,89	91,06	-3,35	12	19699504	+	
cg06007201	-7,55	-311,93	89,29	-2,05	16	88850218	+	*FAM38A*
cg06142740	5,91	-300,47	138,22	-1,01	2	28973577	+	*PPP1CB*
cg06163904	-0,21	-297,36	240,34	-0,87	17	78964779	+	*CHMP6*
cg06240854	-2,73	-217,23	145,73	4,17	2	99771011	-	*LIPT1*
cg06247837	-4,13	-201,15	168,69	2,28	17	37820135	-	*TCAP*
cg06285727	35,75	-213,65	86,43	-6,26	11	72524028	+	*ATG16L2*
cg06413398	-11,54	-137,38	97,89	-0,39	6	110736865	+	*DDO*
cg06437747	-8,05	-222,86	119,46	-1,06	1	2890836	-	
cg06484360	-9,11	-271,89	86,62	-0,59	19	18434522	-	*LSM4*
cg06567855	-13,18	-338,56	99,61	-2,33	2	85977911	-	
cg06639320	43,26	252,91	-59,85	6,84	2	106015739	-	*FHL2*
cg06647068	-8,45	-197,14	118,13	1,19	12	104853274	-	*CHST11*
cg06661266	5,07	-200,46	162,80	-0,32	3	134027721	-	
cg06685111	-1,22	-323,40	180,94	2,02	6	30295466	-	*HCG18*
cg06777902	4,17	-190,67	96,93	-3,86	10	127622996	-	*FANK1*
cg06819357	0,68	-231,21	198,71	-3,14	14	102928437	+	*TECPR2*
cg06911110	13,08	-200,77	172,98	-0,08	1	17293971	-	*CROCC*
cg06934523	-4,02	-270,76	141,28	-2,81	14	70077930	-	*KIAA0247*
cg07027613	-11,51	-197,46	92,91	5,55	12	7260608	-	*C1RL*
cg07080372	-28,87	-267,00	107,14	4,06	11	796607	-	*SLC25A22*
cg07082267	-19,67	-209,06	142,41	2,05	16	85429035	-	
cg07092212	14,79	-213,04	87,87	-0,03	11	46382544	-	*DGKZ*
cg07127410	-0,92	-177,01	151,78	-2,58	22	29427851	-	*ZNRF3*
cg07164639	-3,45	-125,35	105,16	-0,03	6	110736958	+	*DDO*
cg07191657	4,39	-189,52	137,76	-0,04	10	14478541	+	*MIR1265*
cg07211259	-20,47	-186,97	93,34	-3,15	9	5510497	-	*PDCD1LG2*
cg07234388	7,10	-276,88	231,46	-4,10	1	156261841	+	*TMEM79*
cg07368443	-6,85	-295,40	166,98	-0,73	1	45265337	+	*PLK3*
cg07388493	0,73	-192,70	144,03	-2,32	1	39491459	-	*NDUFS5*
cg07568841	-6,12	-191,56	103,13	-1,15	7	30362781	+	*ZNRF2*

cg07583137	-11,28	-205,73	192,32	0,60	8	82644012	-	*CHMP4C*
cg07703358	5,38	-259,01	95,15	3,29	7	138702117	-	
cg07843120	-17,70	-264,09	86,28	1,03	19	34856957	-	*GPI*
cg07850154	0,52	-191,20	125,42	2,84	5	63461232	+	*RNF180*
cg07931844	-9,24	-256,80	146,83	-0,96	15	72102213	-	*NR2E3*
cg08090640	0,75	-219,26	157,99	4,61	17	41159289	-	*IFI35*
cg08128734	-8,44	-156,31	138,19	-2,66	1	206685423	-	*RASSF5*
cg08138505	-18,03	-353,07	100,07	4,99	11	67118746	-	*LOC1001309*
cg08160331	3,43	274,45	-76,25	-3,16	11	75140865	+	*KLHL35*
cg08234504	-13,07	-279,76	137,63	0,68	5	139013317	+	
cg08262002	4,14	-184,95	151,48	-0,48	4	16575323	+	*LDB2*
cg08301612	4,68	-175,99	109,32	0,66	17	43228571	+	*HEXIM1*
cg08337633	22,78	-385,83	83,83	5,51	7	55602109	-	*VOPP1*
cg08343101	18,77	-290,94	183,96	0,09	11	44933332	-	*TSPAN18*
cg08409562	11,88	-220,04	168,08	0,10	6	31737885	-	*C6orf27*
cg08415592	2,45	-228,55	113,12	6,45	22	36648973	-	*APOL1*
cg08426733	-12,73	-306,81	168,88	0,63	1	1376740	-	*VWA1*
cg08453194	9,26	-314,30	180,23	0,94	6	41904398	+	*CCND3*
cg08468689	-3,99	-254,36	113,24	-1,37	17	40346680	-	*GHDC*
cg08471846	20,47	-267,28	132,19	0,96	19	799621	-	*PTBP1*
cg08511485	-1,25	-252,56	90,62	1,30	3	168945731	-	*MECOM*
cg08541155	-5,90	-188,63	113,25	0,16	3	118994052	+	
cg08570034	-3,07	-166,10	141,42	-2,35	5	175227702	+	*CPLX2*
cg08644498	16,85	-318,24	188,11	-1,21	1	46502608	+	
cg08713098	-19,92	-264,02	114,70	-1,41	7	100005500	-	*ZCWPW1*
cg08761208	-12,68	-309,43	119,55	-5,14	15	65693289	+	*IGDCC4*
cg08784091	20,57	-436,82	108,76	-2,01	6	31670005	-	*BAT5*
cg08877357	2,23	-136,48	94,70	2,80	10	113120532	+	
cg08913523	-3,98	-189,17	93,39	1,39	8	126649807	+	
cg08957484	12,33	198,18	-25,13	6,49	5	132083532	+	*CCNI2*
cg09124496	-1,93	-176,00	164,47	3,01	7	41735851	+	*LOC285954*
cg09143195	-11,29	-162,82	88,94	-4,66	12	41085901	-	*CNTN1*
cg09294739	-10,31	-147,58	79,27	0,80	16	55218782	-	
cg09308553	1,56	-280,58	157,71	-1,63	16	56763573	-	*NUP93*
cg09340639	-2,50	-231,57	84,57	2,12	1	157789662	-	*FCRL1*
cg09608765	5,19	-266,54	120,69	0,79	3	45636137	+	*LIMD1*
cg09642020	-7,43	-311,17	201,35	-1,71	9	139379173	+	*C9orf163*
cg09748749	-11,67	-228,12	145,98	1,62	7	65540429	+	*ASL*
cg09809672	-11,12	-163,69	131,16	3,87	1	236557682	-	*EDARADD*
cg10052840	-13,04	-265,32	201,66	-0,84	19	4558119	-	*SEMA6B*
cg10137837	12,88	245,62	-34,67	3,56	17	6926742	+	*BCL6B*
cg10448052	12,43	-223,96	90,50	-4,44	12	53715787	+	*AAAS*
cg10476085	19,04	-259,86	175,31	1,33	5	142065737	-	*FGF1*
cg10650821	-6,72	-342,75	105,94	0,68	6	31543686	+	*TNF*
cg10717214	4,33	-315,76	100,66	1,53	6	31543557	-	*TNF*
cg10986043	6,41	-226,08	171,09	0,97	17	37820495	-	*TCAP*
cg11076306	-9,99	-193,78	177,06	-4,53	4	41430667	+	*LIMCH1*
cg11142333	7,79	-275,46	239,90	3,32	1	173840261	+	*ZBTB37*
cg11194994	0,72	-296,60	134,06	-3,59	1	160175974	-	*PEA15*
cg11331344	7,19	-288,68	143,33	0,50	17	73629463	-	*RECQL5*
cg11344352	-7,29	-366,89	70,09	2,15	19	45927696	+	*ERCC1*
cg11436113	-4,31	-234,05	189,57	2,44	20	19191145	-	
cg11453058	-2,97	-260,29	104,34	-2,18	19	14064176	-	*PODNL1*
cg11619216	-3,65	-258,29	213,67	0,17	17	73642080	-	*RECQL5*

cg11741201	12,82	-210,59	102,49	0,69	11	35638398	-	*FJX1*
cg11807280	-2,14	-120,85	111,53	0,26	2	66654644	+	
cg11826475	13,59	-299,07	156,00	1,68	12	120704006	-	*PXN*
cg11836829	-3,16	-251,32	115,15	1,31	1	212605702	-	*NENF*
cg12009872	7,26	-269,47	113,12	-2,21	15	51520739	-	*CYP19A1*
cg12068124	-4,41	-289,10	104,00	4,15	19	1238538	+	*C19orf26*
cg12079303	-8,01	-175,75	118,32	2,06	1	61547163	-	*NFIA*
cg12179661	16,66	-269,98	194,66	7,92	9	140333805	+	*ENTPD8*
cg12197142	13,30	-315,66	115,96	-0,70	17	73629245	-	*RECQL5*
cg12261786	-11,36	-256,31	233,07	-7,12	10	88727830	+	*C10orf116*
cg12278474	13,28	-206,55	117,51	0,51	1	5221357	+	
cg12283460	-0,15	-247,74	94,11	1,71	15	59279556	+	*RNF111*
cg12483947	-14,22	-208,51	111,56	0,41	10	72640100	-	*SGPL1*
cg12526474	7,94	-336,58	125,92	0,08	7	140097579	-	*SLC37A3*
cg12554573	-5,90	-307,33	254,28	-6,71	3	51976667	-	*PARP3*
cg12623930	5,33	-324,35	129,20	0,80	3	52008802	-	*ABHD14B*
cg12681001	12,46	-322,11	95,77	-5,15	6	31543540	-	*TNF*
cg12688670	27,75	-322,50	134,27	-0,01	16	29801602	+	*KIF22*
cg12706425	1,95	-221,56	90,84	-2,62	9	73029578	+	*KLF9*
cg12899747	-13,84	-168,66	103,65	0,85	3	25391527	-	
cg13033938	-9,09	-428,42	80,91	5,59	3	49824475	-	*IP6K1*
cg13055199	4,94	-203,19	147,42	-7,21	15	75136278	-	*ULK3*
cg13066481	16,36	-228,44	161,87	-3,76	3	123372199	-	*MYLK*
cg13072940	8,69	-256,79	143,29	-3,27	3	49967521	+	*MON1A*
cg13120986	1,71	-189,15	100,79	-4,51	12	7168012	-	*C1S*
cg13203811	40,78	-254,49	164,10	-1,88	12	58136245	-	*AGAP2*
cg13319938	-25,50	-321,39	257,70	-6,83	11	2436251	-	*TRPM5*
cg13385220	7,87	-194,42	175,39	-1,56	1	202250453	+	*LGR6*
cg13420364	16,80	-157,69	120,15	0,34	1	234857659	-	
cg13428009	-13,91	-371,17	89,29	-1,74	9	139383171	-	
cg13458211	-2,82	-263,78	172,52	-5,73	1	44444217	+	*B4GALT2*
cg13501527	12,33	-292,84	211,66	3,66	9	123640474	-	*PHF19*
cg13640414	-0,33	-203,94	131,41	2,75	19	15530870	+	*AKAP8L*
cg13683374	-3,27	-235,36	138,93	-0,44	17	72364767	-	*GPR142*
cg13709639	-3,68	-204,32	83,91	-1,88	12	49526040	+	*TUBA1B*
cg13790426	13,23	-219,60	82,78	1,34	1	109506963	-	*CLCC1*
cg13807549	-3,74	-220,42	129,11	3,45	9	116444721	-	
cg13823169	5,90	-247,99	171,51	0,97	9	139776893	-	
cg14039301	-9,69	-296,07	217,98	4,24	11	296540	+	
cg14042143	-2,02	-191,94	183,64	0,92	7	2646782	-	*IQCE*
cg14058848	-17,14	-258,49	202,96	1,20	9	130479114	-	*TTC16*
cg14175438	-8,66	-260,71	107,65	8,26	7	121036729	+	*FAM3C*
cg14188401	-8,95	-296,18	256,90	0,75	3	134097684	-	
cg14292522	8,73	-183,12	121,91	2,70	12	100535489	+	*UHRF1BP1*
cg14305711	-3,85	-225,88	88,10	1,42	2	62132430	+	*COMMD1*
cg14314729	3,51	-225,50	146,02	-0,29	5	179815975	-	
cg14359680	-20,10	-272,20	121,60	1,72	14	105532208	-	*GPR132*
cg14583999	-6,02	-142,51	124,39	1,38	3	10019040	-	*TMEM111*
cg14609289	7,37	-318,36	226,19	0,66	17	77083037	+	*ENGASE*
cg14671809	14,85	-231,07	183,27	0,73	3	55558213	-	*ERC2*
cg14775286	10,45	-203,10	131,28	3,11	3	137833494	+	*DZIP1L*
cg14837598	-10,15	-220,67	191,64	1,59	8	21916635	-	*EPB49*
cg14898223	-0,49	-147,98	123,86	2,32	2	190447407	+	
cg14934280	31,20	-285,25	120,40	3,58	19	47494587	+	*GRLF1*

cg14956327	9,60	-134,42	96,87	1,67	6	110737053	-	*DDO*
cg14963724	-6,40	-222,69	79,36	6,84	18	72166303	-	*CNDP2*
cg14973055	11,24	-176,27	138,41	5,80	17	72306038	-	*DNAI2*
cg14977938	6,85	-258,46	231,65	-1,72	14	104190829	+	*ZFYVE21*
cg14989226	15,51	-219,92	118,18	-0,31	7	75897850	-	*SRRM3*
cg15034393	28,13	-150,56	101,63	0,89	3	152856773	-	
cg15125438	-7,91	-293,54	255,00	-1,88	12	113684389	+	*TPCN1*
cg15298486	10,19	-274,12	99,10	-5,76	10	102046690	+	*BLOC1S2*
cg15393702	3,30	-191,53	152,34	-0,39	18	21243529	+	*ANKRD29*
cg15416179	-26,46	-310,10	71,25	4,35	17	21189859	-	*MAP2K3*
cg15538427	0,56	-284,81	203,20	-1,26	11	62457014	-	*LRRN4CL*
cg15704699	13,31	-230,64	203,01	3,72	17	26439377	+	*NLK*
cg15743533	22,43	-262,75	100,05	7,50	20	815345	+	*FAM110A*
cg15804973	1,55	-177,55	118,91	-0,92	6	137114513	+	*MAP3K5*
cg15829826	3,74	-411,22	87,36	1,61	11	65153860	+	*FRMD8*
cg15845821	16,65	-192,41	141,59	0,81	19	16830613	-	*NWD1*
cg15893346	3,66	-224,32	131,55	2,16	7	65447565	+	*GUSB*
cg15903032	-6,50	-228,96	161,30	2,26	10	101297605	-	
cg16193278	-7,16	-264,85	103,98	0,54	13	100008450	-	*UBAC2*
cg16273597	-0,68	-273,88	103,17	3,75	6	14117480	+	*CD83*
cg16276108	-5,36	-226,19	143,76	0,19	5	180213189	+	
cg16363586	7,30	-241,65	91,83	-0,51	19	17516329	-	*BST2*
cg16541026	-2,21	-236,27	145,10	0,31	3	49026934	-	*P4HTM*
cg16618104	6,08	-252,79	103,05	-2,10	12	104853100	+	*CHST11*
cg16640358	-5,66	-279,38	150,10	1,20	2	239892057	-	
cg16664617	-14,33	-293,24	249,71	-3,10	19	17607007	-	*SLC27A1*
cg16677191	-16,19	-191,76	98,02	-1,25	5	95159423	-	*GLRX*
cg16742481	-14,93	-246,35	155,94	-0,49	4	157090555	-	
cg16744741	-1,77	-161,72	117,41	2,10	4	82126025	-	*PRKG2*
cg16762684	17,79	-198,51	69,02	3,01	18	74820493	+	*MBP*
cg16810343	3,09	-244,57	136,21	-0,50	7	75898970	+	*SRRM3*
cg16867657	48,86	173,46	-55,16	5,22	6	11044877	+	*ELOVL2*
cg16960758	26,38	-198,22	165,68	-0,99	2	169658992	-	*NOSTRIN*
cg16983588	-6,27	-157,18	127,01	-0,09	11	129793108	+	*PRDM10*
cg17133388	13,00	-357,00	98,64	1,74	3	122102727	+	*FAM162A*
cg17168836	-15,50	-191,31	115,17	1,29	1	68256161	-	*GNG12*
cg17183905	-26,14	-242,45	107,33	-0,34	12	110253842	-	*TRPV4*
cg17329534	-1,71	-228,74	99,00	0,04	1	154980743	-	*ZBTB7B*
cg17436656	2,13	-231,24	174,78	-0,03	12	53627106	+	*RARG*
cg17457912	5,23	-228,33	161,74	-6,58	17	1617102	-	*C17orf91*
cg17593342	4,74	-208,72	177,84	-4,86	6	14037614	-	
cg17621438	-0,71	-202,33	116,50	-1,02	5	63461216	+	*RNF180*
cg17721618	-18,30	-322,23	176,56	6,31	15	42376692	-	*PLA2G4D*
cg18076651	-9,90	-347,32	132,87	-1,46	12	53625605	-	*RARG*
cg18079948	3,55	-134,09	93,42	-1,53	10	20009221	-	
cg18150280	8,53	-202,25	107,78	7,73	1	192776859	-	*RGS2*
cg18186343	-8,02	-179,85	118,20	3,05	14	101317620	-	*MIR770*
cg18215449	-6,83	-177,35	131,88	6,53	12	66089473	+	
cg18333339	8,15	-231,73	173,44	-0,32	3	112885856	-	
cg18450254	-6,16	-126,82	94,13	-1,28	3	64200005	+	*PRICKLE2*
cg18505959	-21,46	-387,82	112,64	-0,35	6	31670195	-	*BAT5*
cg18568843	-13,99	-353,69	110,82	-0,94	6	52535550	+	*TMEM14A*
cg18651026	-10,21	-270,92	203,20	2,32	6	33140660	-	*COL11A2*
cg18715243	-1,80	-262,98	221,76	1,19	8	37658755	-	*GPR124*

cg18738190	14,72	-153,90	132,31	-0,37	10	73740291	+	*CHST3*
cg18779283	-14,71	-365,20	107,52	0,49	3	45636154	+	*LIMD1*
cg18786171	9,15	-248,96	157,65	1,85	10	75935758	+	*ADK*
cg18797590	0,18	-191,60	164,95	7,38	4	15480643	-	*CC2D2A*
cg18808904	-2,59	-194,23	137,47	-3,05	20	11898851	-	*BTBD3*
cg18826637	-9,64	-138,02	123,75	1,89	2	145116633	+	
cg18887458	3,91	-114,39	85,51	6,05	7	115995934	+	
cg18933331	-6,38	-210,85	151,07	2,60	1	110186418	-	
cg18993949	5,35	-263,76	203,05	-0,01	4	15481269	+	*CC2D2A*
cg19025497	-9,54	-284,05	97,12	-0,79	5	138534324	+	*SIL1*
cg19056004	10,84	-227,46	173,68	0,52	12	7023262	-	*LRRC23*
cg19261426	-2,63	-209,97	171,24	-2,16	1	228560439	+	*OBSCN*
cg19270739	20,34	-181,04	125,21	-1,63	1	1368846	-	
cg19344626	-16,80	-160,82	164,61	3,47	19	16830749	-	*NWD1*
cg19381811	-5,63	-190,15	103,44	3,28	3	49851713	-	*UBA7*
cg19433091	17,07	-253,45	120,00	3,23	15	43426498	-	*TMEM62*
cg19453093	-0,07	-197,53	140,02	-0,75	14	88655985	+	*KCNK10*
cg19500607	-5,25	-243,29	205,69	3,80	5	148034319	+	*HTR4*
cg19578183	-7,97	-265,46	207,95	0,10	2	128991530	+	
cg19612068	23,19	-248,98	158,25	-0,63	6	33130024	+	
cg19670290	-7,57	-227,09	135,21	1,24	15	91477210	-	*HDDC3*
cg19722847	-4,47	-266,35	98,96	0,46	12	30849114	+	*IPO8*
cg19729744	10,11	-151,66	134,86	-1,78	3	194752020	+	
cg19753867	18,32	-279,61	139,57	-1,15	20	35383166	-	*DSN1*
cg19758448	-0,29	-169,21	145,82	-2,86	17	37828296	+	*PGAP3*
cg19761273	20,53	-261,03	123,42	0,83	17	80232096	+	*CSNK1D*
cg19784428	13,77	-185,42	179,50	3,66	19	16830746	-	*NWD1*
cg19804488	-3,56	-269,52	187,29	-6,29	17	73760363	-	*GALK1*
cg19838043	14,44	-211,09	190,40	0,53	14	104196038	-	*ZFYVE21*
cg19936954	7,20	-259,98	124,24	-2,11	1	180148192	-	*QSOX1*
cg20052760	3,07	-210,40	109,64	-1,06	6	10510789	+	
cg20067719	0,58	-198,86	110,13	0,55	17	48623772	+	*SPATA20*
cg20133890	-3,05	-217,05	159,64	3,36	6	31680144	-	*LY6G6E*
cg20153322	-20,16	-245,84	187,60	0,53	12	120703977	-	*PXN*
cg20222376	0,99	-191,10	89,01	-1,63	19	15530606	-	*AKAP8L*
cg20249566	-10,57	-182,93	176,09	3,70	19	16830739	-	*NWD1*
cg20267101	2,65	-199,50	142,41	-6,42	3	113006232	-	*BOC*
cg20303331	-10,33	-234,48	157,21	-0,66	17	36945575	-	*PIP4K2B*
cg20515136	4,47	-226,62	133,18	-4,81	3	159707145	-	*IL12A*
cg20543183	-19,01	-277,48	211,44	1,81	6	31648146	+	*LY6G5C*
cg20595453	-18,16	-227,30	187,71	-0,17	6	33219392	-	*VPS52*
cg20608990	-1,99	-199,22	124,16	1,50	2	202097607	+	*CASP8*
cg20669012	-6,95	-249,98	124,96	-3,62	3	11102341	+	
cg20747538	-12,77	-247,03	130,92	1,12	3	137838021	+	
cg20813374	5,53	-253,22	161,81	-0,33	6	35657180	-	*FKBP5*
cg20822990	-12,75	-270,87	143,67	-1,78	1	17338766	-	*ATP13A2*
cg20912205	4,02	-265,50	121,18	-2,05	3	50337305	-	*NAT6*
cg20964856	4,24	-271,65	182,64	-1,20	19	41767669	-	*HNRNPUL1*
cg20988565	-8,00	-171,10	142,18	-0,85	8	106334333	+	*ZFPM2*
cg21120249	-8,51	-233,89	191,87	2,33	9	139921971	-	*C9orf139*
cg21139312	8,12	300,34	-217,34	3,88	17	55663225	-	*MSI2*
cg21186955	-2,39	-224,32	118,83	1,70	7	100729412	-	*TRIM56*
cg21222743	-6,84	-307,87	88,93	0,64	6	31543545	-	*TNF*
cg21280392	-0,28	-232,27	158,20	-2,36	17	47304116	+	*PHOSPHO*[illegible]

cg21322248	-55,50	-376,28	85,78	0,52	15	77289047	-	*PSTPIP1*
cg21333674	-3,48	-138,10	112,71	1,00	8	96705765	-	
cg21406967	-8,98	-235,14	141,98	0,87	7	100464553	-	*TRIP6*
cg21469505	10,81	-195,72	122,74	-0,06	18	47085749	-	
cg21523751	2,02	-180,70	94,77	-0,30	1	182988639	+	
cg21524899	9,29	-325,95	279,38	-0,27	1	225662606	-	
cg21554217	1,58	-295,31	107,96	-0,47	5	138897467	-	
cg21572722	-9,77	333,99	-87,27	6,39	6	11044894	+	*ELOVL2*
cg21635307	-10,63	-268,86	105,08	-2,04	6	90319877	+	*ANKRD6*
cg21868031	-33,79	-387,58	90,18	1,09	1	207925112	-	*CD46*
cg21878650	-3,96	-110,20	82,54	0,43	5	64558623	+	*ADAMTS6*
cg21922223	-8,72	-343,10	106,99	3,90	17	75539118	+	
cg21940640	5,65	-210,38	173,54	-0,67	7	56160893	+	*PHKG1*
cg21962791	1,17	-204,91	134,47	-2,33	12	21590305	-	*PYROXD1*
cg21990700	-2,63	-207,22	108,02	-1,91	12	7260776	-	*LOC283314*
cg22156456	17,40	-247,48	108,72	1,54	17	39844239	-	*EIF1*
cg22156842	-1,61	-172,15	101,14	0,72	3	136537169	-	*TMEM22*
cg22273555	1,86	-231,68	137,81	5,00	6	33130034	+	
cg22299097	-11,98	-203,49	113,38	1,86	3	97690590	-	*MINA*
cg22361181	-19,18	-290,19	174,24	-3,05	17	40171740	-	*NKIRAS2*
cg22379463	-15,89	-253,37	192,09	-1,92	3	45260330	-	
cg22454769	1,25	149,85	-29,55	8,85	2	106015767	-	*FHL2*
cg22584802	3,55	-214,53	91,14	-3,27	7	129007902	-	*AHCYL2*
cg22730004	-3,80	-140,52	93,22	1,84	1	158656718	-	*SPTA1*
cg22737154	-25,64	-151,96	123,69	-1,97	2	64631614	-	
cg22747507	8,89	-235,50	161,36	4,83	4	175635801	-	*GLRA3*
cg22768222	5,48	-146,75	111,32	-9,10	6	45383690	+	*RUNX2*
cg22864266	8,16	-354,17	115,85	1,30	12	49319673	-	*FKBP11*
cg22927302	-27,38	-312,35	222,58	3,02	3	50304463	-	*SEMA3B*
cg22929506	-10,00	-214,65	123,43	2,14	2	219137490	-	*PNKD*
cg22947000	9,74	-171,18	118,64	0,59	16	81272281	-	*BCMO1*
cg22976533	3,86	-189,14	139,90	0,78	14	105784090	-	*PACS2*
cg23003085	-0,25	-228,96	93,30	-4,20	11	64084321	-	*PRDX5*
cg23078123	-13,92	-232,69	187,66	7,08	1	68577796	+	*GPR177*
cg23124451	8,67	-268,34	184,82	1,64	22	39548131	+	*CBX7*
cg23149300	0,71	-239,89	125,37	0,40	10	36958940	+	
cg23149687	8,36	-166,55	90,20	-5,17	5	119801643	+	*PRR16*
cg23320649	1,58	-255,59	185,01	0,56	3	50604613	+	*C3orf18*
cg23341182	-11,30	-198,43	117,26	2,06	10	102046768	+	*BLOC1S2*
cg23368715	1,06	-222,64	153,53	3,01	12	7245510	-	*C1R*
cg23389651	4,65	-334,15	107,12	0,11	13	95327390	+	
cg23460961	-4,53	-163,74	95,77	-1,51	11	94382347	+	
cg23461714	-22,42	-293,77	109,35	-0,74	11	113184990	-	*TTC12*
cg23677833	-17,05	-261,23	95,68	0,21	19	16308345	-	*AP1M1*
cg23715749	14,85	-187,19	130,80	-1,53	1	37413867	+	*GRIK3*
cg23718736	-2,67	-217,04	166,91	1,47	18	6413908	-	*L3MBTL4*
cg23732483	-5,71	-163,08	87,48	1,41	3	48965611	+	*ARIH2*
cg23744638	-12,12	-177,22	173,67	1,61	11	10323902	-	
cg23836737	5,97	-237,23	135,86	1,07	4	53411703	+	
cg23950157	-15,21	-288,76	115,13	-6,13	17	48275919	+	*COL1A1*
cg23972551	-9,52	-360,46	102,49	1,05	11	68151664	-	*LRP5*
cg24057710	-1,55	-253,92	184,59	2,76	11	126311095	+	*KIRREL3*
cg24079702	-1,04	197,30	-21,96	7,32	2	106015771	-	*FHL2*
cg24155190	0,12	-303,80	89,83	-0,80	1	201476619	-	*CSRP1*

cg24711336	4,67	-254,99	198,51	4,19	10	80063791	-	
cg24774812	-8,42	-280,50	102,77	1,96	14	63743552	-	*RHOJ*
cg24847230	29,00	-345,09	194,89	1,35	17	46986807	-	*UBE2Z*
cg24848615	-4,31	-220,80	187,81	0,61	19	3368396	-	*NFIC*
cg24883498	0,81	-264,05	120,12	-0,54	19	18264771	+	*PIK3R2*
cg24892069	1,73	-122,69	118,06	4,72	10	33562205	-	*NRP1*
cg24987259	-0,29	-223,14	103,71	3,30	11	66336293	-	*CTSF*
cg25150953	3,41	-126,31	77,61	1,53	4	41540229	-	*LIMCH1*
cg25268718	14,86	-271,51	203,37	-1,91	14	24604711	+	*PSME1*
cg25311470	4,51	-233,58	122,80	-4,02	7	107950866	+	*NRCAM*
cg25371036	-3,41	-231,10	202,60	-0,16	11	94500749	-	*AMOTL1*
cg25395188	9,46	-251,24	95,73	0,12	11	60897782	+	*VPS37C*
cg25413977	2,33	-145,21	127,85	-8,32	2	66651619	+	
cg25424279	0,23	-260,30	233,41	0,14	11	65683543	+	
cg25439632	4,21	-140,47	86,99	3,08	8	49892209	+	
cg25616535	-5,06	-294,17	185,38	0,34	17	47294967	-	*ABI3*
cg25782440	-17,64	-334,88	238,90	-1,23	19	7979022	-	*MAP2K7*
cg25793051	2,45	-229,49	112,53	2,30	7	149439005	-	
cg25994988	-13,38	-237,33	166,82	1,40	11	122652382	+	*UBASH3B*
cg25998745	11,51	-217,03	190,09	1,77	8	142028625	+	
cg26094232	11,09	-228,96	132,93	-1,30	11	72148947	+	
cg26101277	-17,16	-202,69	89,03	-1,13	17	32690412	-	*CCL1*
cg26158023	-8,76	-326,01	128,46	3,01	3	42881414	+	*CCBP2*
cg26166595	3,08	-211,39	128,53	2,78	11	11998715	+	*DKK3*
cg26210267	-3,56	-303,00	118,61	1,68	4	668877	+	*ATP5I*
cg26276120	4,90	-312,80	116,93	5,26	12	6977747	-	*TPI1*
cg26290219	-12,78	-216,50	156,60	-3,10	6	33128906	-	
cg26316599	8,43	-207,54	154,55	-2,14	5	172456055	+	*ATP6V0E1*
cg26373518	8,52	-216,11	138,73	-3,12	22	31518942	-	*INPP5J*
cg26450750	-5,18	-271,34	107,17	-2,32	4	87816983	-	
cg26483332	-14,07	-348,01	115,43	-1,60	10	76993756	+	*COMTD1*
cg26543112	-12,35	-234,51	107,03	0,34	6	133188277	+	
cg26608718	-8,45	-153,44	100,70	-1,62	19	15530737	-	*AKAP8L*
cg26610808	-20,20	-291,32	100,24	1,58	10	102046685	+	*BLOC1S2*
cg26614073	-5,59	-211,36	152,16	2,72	3	47517819	-	*SCAP*
cg26748477	0,81	-252,00	206,70	0,46	17	38516415	+	
cg26787199	15,94	-359,48	101,35	-0,42	16	2044042	-	*SYNGR3*
cg26808293	-0,47	-434,47	132,06	-4,27	16	3072207	-	*TNFRSF12*
cg26894354	-0,65	-228,87	143,90	-1,40	1	203311314	-	*FMOD*
cg26954174	1,19	-168,23	87,32	0,85	16	50730813	-	*NOD2*
cg26963632	4,50	-196,16	103,48	-0,36	16	85558148	-	
cg26969888	0,66	-201,74	150,42	-0,06	19	14064254	-	*PODNL1*
cg27004870	10,10	-328,68	100,33	3,05	16	88850384	+	*FAM38A*
cg27209729	-6,92	-172,72	143,58	-0,40	11	64428925	+	*NRXN2*
cg27236973	2,57	-160,22	95,13	-4,11	17	39781997	+	*KRT17*
cg27259408	-2,44	-258,10	183,70	3,73	19	10427154	+	*FDX1L*
cg27269561	-11,57	-339,78	132,23	-2,48	16	3072713	+	*HCFC1R1*
cg27346545	-5,29	-200,51	132,47	-7,38	20	1205378	-	*RAD21L1*
cg27386529	3,29	-227,37	142,67	0,70	3	47517807	-	*SCAP*
cg27401724	-7,04	-272,41	215,54	-3,82	17	43213629	-	*ACBD4*
cg27409484	1,61	-261,72	96,08	-2,41	6	71721893	+	
cg27470213	13,46	-264,14	91,01	1,35	17	76967695	-	*LGALS3BP*
ch.1.171672612F	-38,90	-347,67	81,10	-3,68	1	173405989	+	
ch.1.839062R	-5,65	-333,21	82,11	0,72	1	25282539	+	*RUNX3*

ch.13.39564907R	22,73	-308,24	77,43	1,80	13	40666907	+
ch.14.97331099F	72,73	-394,17	71,25	-3,05	14	98261346	+
ch.15.67797584R	-10,47	-236,06	84,19	0,60	15	70010530	+
ch.19.16251119F	-12,63	-346,33	71,26	-0,69	19	16390119	+
ch.2.105901354F	-0,63	-233,23	72,08	0,39	2	106534922	+
ch.2.207814544R	2,99	-257,09	71,31	1,39	2	208106299	+
ch.2.217478R	-4,18	-367,81	68,03	2,12	2	8142529	+
ch.2.30415474F	-22,63	-289,72	77,83	-1,34	2	30561970	+
ch.2.47286786F	6,34	-448,44	71,05	0,65	2	47433282	+
ch.6.33611621F	-9,61	-328,25	75,08	0,34	6	33503643	+

For each of the 491 methylation sites, we include the coefficients for the multivariate model, the slopes and intercepts for the average of n-independent linear regressions, and the weights for the WKDE model.

Table S8. Pyrosequencing data used for the 9CpG WKDE model.

		FHL2-2	*IGSF11-1*	*CCDC-2*	*MEIS1-1*	*ELOVL2-4*	*COL1A1-1*	*PDE4C-1*	*ASPA-1*	*ITGAB-2*	Real Age
	**Weight (WKDE)**	6,2	-1,4	5,6	-2,3	9,6	0,4	3,5	2,1	4,2	-
	**Sample ID**										
Training	K	23,46	59,42	35,34	57,91	33,31	33,46	14,47	83,19	66,87	19
	U	23,33	45,06	33,1	49,11	4,01	35,54	13,02	75,48	41,15	21
	BR	30,04	52,67	30,43	51,13	25,26	37,27	14,84	72,12	59,15	23
	BK	31,4	54,67	31,25	39,61	39,09	44,19	20,8	68,36	62,29	24
	AE	34,91	46,01	29,15	40,36	23,01	30,31	18,63	71,48	54,47	26
	S	33,62	49,31	32,64	47,79	25,7	42,83	14,34	67,99	53,7	27
	CP	37,89	44,85	28,38	26,82	35,99	23,6	21,66	69,06	61,26	29
	Z	30,31	52,65	24,65	42,64	18,56	31,48	17,56	64,86	56,18	30
	CD	37,58	45,58	31,86	43,6	44,76	40,76	19,56	73,06	66,38	33
	CS	35,24	38,24	33,59	47,84	28,15	43,14	18,59	72,34	49,81	34
	AN	37,5	31,82	27,53	31,55	37,9	26,87	19,94	64,37	66,26	35
	AS	36,48	40,51	29,94	40,24	35,37	36,69	17,78	70,57	50,31	37
	CK	41,68	22,91	28,88	27,96	46,58	30,31	25,2	77,84	54,28	41
	Y	39,82	29,98	23,92	26,66	46,54	22,8	19,33	70,67	62,97	42
	CH	43,02	26,42	24,8	24,59	49,6	29,14	20,37	74,18	61,59	44
	AA	35,29	53,82	21,53	24,73	8,9	26,45	22,95	59,26	33,31	44
	BL	35,08	31,77	24,69	20,91	54,34	22,38	20,8	67,83	64,73	47
	AB	30,64	46,33	18,73	31,33	52,33	30,92	19,27	65,73	45,51	47
	CJ	47,09	19,67	17,03	17,32	49,61	29,99	26,56	56,87	50,42	50
	BV	42,22	40,2	26,52	31,62	35,47	30,2	29,46	63,45	49,1	51
	BS	44,33	38,26	32,66	31,67	64,37	36,05	21,55	48,82	57,51	53
	AT	44,41	19,09	18,29	23,75	44,38	27,49	27,17	50,21	58,2	54
	AQ	55,75	19,04	23,01	27,45	41,95	20,94	26,84	60,54	53,08	56
	AC	48,66	23,1	22,33	26,53	51,39	21,01	32,52	67,6	64,85	57
	AW	39,2	37,63	14,4	26,49	43,24	20,55	27,02	71,3	56,25	59
	CR	52,16	18,98	18,29	18,59	72,03	24,89	23,31	50,02	52,12	60
	AF	50,13	21,09	20,31	16,34	70,57	24,09	29,11	68,07	52,45	62
	AL	54,83	20,21	18,21	17,57	53,67	20,47	26,3	55,55	58,18	63
	BA	46,64	20,22	20,25	18,99	40,44	31,14	37,83	67,81	47,55	65
	H	51,29	34,04	11,16	28,39	66,38	18,32	28,64	59,72	45,01	66
	AP	56,84	16,9	18,43	25,71	59,43	22,79	30,84	72,49	58,07	72
	AO	39,99	36,23	9,24	18,52	72,44	20,88	33,13	52,51	37,76	73
Validation	X	37,02	50,71	32,75	35,68	30,13	30,17	15,07	76,19	63,68	21
	BC	33,57	62,83	38,7	54,36	28,55	36,9	16,85	81,24	71,09	21
	BI	34,13	57,36	32,79	40,57	32,17	33,47	14,85	79,68	55,3	21
	BD	28,16	53,06	33,33	44,16	33,2	28,38	13,37	73,22	51,98	22
	BN	29,51	51,7	32,68	51,72	35,72	29,58	14,15	77,45	67,11	22
	BM	36,43	39,3	34,83	76,18	45,92	28,2	16,26	78,37	57,99	23
	BP	30,82	48,01	30,38	31,5	22,43	30,97	17,84	70,51	57,42	25
	BG	32,32	49,9	29,93	35,21	50,07	30,63	14,91	82,72	69,55	27
	BY	38,28	29,74	31,64	35,05	37,25	30,19	16,13	76,25	48,92	27
	CG	33,19	38,53	31,24	45,19	38,91	31,98	11,55	76,13	62,3	27
	CO	28,68	39,56	29,28	44,38	29,43	26,18	19,56	83,21	60,03	27
	AX	38,97	28,7	29,25	48,98	24,47	23,79	14,04	79,97	47,77	29
	BZ	38,13	37,82	28,22	40,28	33,81	37,05	13,17	74,6	51,95	29
	AU	33,36	24,8	28,24	33,33	26,19	30,68	16,02	73,3	61,24	30

AZ	37,55	51,38	33,3	39,98	44,21	31,17	16,03	74,32	62,68	30
BB	36,86	44,58	29,45	34,38	31,05	26,35	17,01	71,17	55,35	30
AV	40,25	44,6	28,85	32,22	55	30,41	17,11	74,45	47,68	31
BW	42,32	33,89	24,84	51,81	46,5	28,98	19,11	73,46	55,91	38
AG	41,87	24,36	21,47	28,09	59,58	22,95	27,9	74,19	57,68	43
BT	42,26	27,82	32,33	38,99	40,66	30,6	29,32	74,89	59,94	43
AY	39,38	25,4	24,84	38,62	61,49	24,73	21,89	71,15	63,1	46
AM	47,25	14,33	9,27	11,56	68,37	28,12	22,87	56,61	47,11	50
BO	45,35	14,78	9,74	18,02	57,02	15,61	28,93	50,37	65,48	50
BU	46,79	33,49	23,35	29,97	50,75	25,79	19,71	77,39	61,37	51
CQ	43,47	24,92	15,88	31,83	48,68	23,05	28,21	57,52	46,51	52
BQ	44,85	18,98	14,84	30,02	49,24	15,98	29,43	49,51	60,36	54
CN	43,82	27,13	25,38	29,5	42,82	24,65	23,36	73,19	51,89	54
AR	51,7	21,66	14,17	21,35	41,63	22,31	33,68	54,9	47,4	57
CM	48,77	20,95	13,24	14,11	61,38	20,12	32,85	43,48	45,63	66
AD	41,79	28,53	27,54	35,34	58,25	26,02	22,57	71,27	62,58	49
AI	36,74	30,91	25,85	48,45	46,59	31,18	17,53	77,6	62,43	28
G	38,83	35,23	27,73	32,2	51,98	27,69	21,91	67,06	57,18	46
N	28,06	48,35	30,67	51,36	22,26	28,71	12,09	74,39	55,4	22
P	55,85	23,05	22,29	14,78	63,12	21,75	32,26	74,84	64,73	65
R	29,31	49,41	32,49	35,84	34,42	30,09	17,94	80,85	65,22	25
Q	33,25	49,41	32,46	49,46	47,17	30,92	18,21	77,41	60,82	31
V	33,04	62,12	32,53	50,54	55,68	40,28	13,55	71,72	52,93	26
BJ	42,96	27,34	26,82	31,34	55,72	30,14	20,26	55,82	48,91	55
BX	43,26	35,24	32,39	37,79	58,6	33,35	22,8	60,35	59,63	40
CB	47,05	44,8	27,76	27,69	60,22	31,08	19,87	60,87	55,08	43
CE	46,42	44,65	22,16	33,26	70,26	33,31	29,01	60,14	64,01	61
CL	45,1	29,79	27,82	26,79	68,03	48,48	21,33	43,66	59,29	52
CI	41,72	31,78	31,69	34,35	24,7	39,24	20,17	56,81	47,91	50
I	50,65	38,17	15,12	16,25	78,67	23,71	28,11	53,2	37,19	64
J	29,53	49,32	25,55	31,74	48,29	31,53	18,33	77,17	45,31	27
L	28,59	39,99	25,11	24,26	11,63	30,33	17,52	58,84	49,17	29
M	27,97	59,09	27,13	36,47	28,17	38,15	16,58	71,34	48,99	23
T	39,05	37,9	19,78	49,26	33,73	28,63	26,63	56,12	38,65	50
